# Comparison of observer based methods for source localisation in complex networks

**DOI:** 10.1038/s41598-022-09031-0

**Published:** 2022-03-24

**Authors:** Łukasz G. Gajewski, Robert Paluch, Krzysztof Suchecki, Adam Sulik, Boleslaw K. Szymanski, Janusz A. Hołyst

**Affiliations:** 1 Center of Excellence for Complex Systems Research, Faculty of Physics, Warsaw University of Technology, Koszykowa 75, 00-662 Warsaw, Poland; 2 Network Science and Technology Center, Rensselaer Polytechnic Institute, 110 8th St, Troy, NY 12180 USA; 3 ITMO University, Kronverkskiy Prospekt 49, Saint Petersburg, 197101 Russia

**Keywords:** Computer science, Complex networks

## Abstract

In recent years, research on methods for locating a source of spreading phenomena in complex networks has seen numerous advances. Such methods can be applied not only to searching for the “patient zero” in epidemics, but also finding the true sources of false or malicious messages circulating in the online social networks. Many methods for solving this problem have been established and tested in various circumstances. Yet, we still lack reviews that would include a direct comparison of efficiency of these methods. In this paper, we provide a thorough comparison of several observer-based methods for source localisation on complex networks. All methods use information about the exact time of spread arrival at a pre-selected group of vertices called observers. We investigate how the precision of the studied methods depends on the network topology, density of observers, infection rate, and observers’ placement strategy. The direct comparison between methods allows for an informed choice of the methods for applications or further research. We find that the Pearson correlation based method and the method based on the analysis of multiple paths are the most effective in networks with synthetic or real topologies. The former method dominates when the infection rate is low; otherwise, the latter method takes over.

## Introduction

In the last decade, there have been numerous advances in the field of locating sources of diffusion in complex networks^1–35^. Now, perhaps more than ever, such studies are relevant to the global challenges our societies face. Whether it is a viral pandemic^36^ or a cascade of misinformation^37^ it can be of utmost importance to ascertain from where the diffusion originated^38–40^. The current literature on this topic is lacking thorough reviews comparing multiple methods of source localisation. Those that do exist^41,42^ are more introductions to the topic than comprehensive comparisons based on computational experiments. While testing all possible variables, scenarios and methods is, of course, practically impossible, we hope that our work will fill in at least some of the gaps.

There are many examples of different approaches of how one can tackle the problem of locating a source of spread in complex networks. Just to name a few, there are works on locating sources on temporal networks^8,17^, using a snapshot of states of the whole system^9^, efficiently placing observers^10,11^ or locating multiple sources^12–16,23^.

In this paper, we consider a graph ${\mathscr {G}}$ with *N* nodes and *E* links. In this graph, one node becomes the *initial source* of a spread of what could be a biological virus or a post on Twitter etc. We assume that we have a certain budget $K < N$ of *observers* that we can place in the graph ${\mathscr {G}}$. Only these observers provide information about the time they received the information propagating through the network. Knowing this limited information and the graph’s topology, we attempt to locate the source using several efficient approaches described recently in the literature (see “Methods” section).

We simulate the spreading phenomenon using the Susceptible-Infected model^43^ that has been one of the most fundamental approaches in this field for almost a century now^44^. In this model, each agent ($n \in {\mathscr {G}}$) can be either Susceptible to or Infected by whatever is diffusing through the system and each Infected node attempts to infect its Susceptible neighbours at each time step. The process is parameterised by an infection rate $\beta$ defining the probability of a Susceptible being infected by one Infected in one time step (see “Methods” section for details).

The goal of this paper is to study the combinations of various observer placement strategies and source location methods while varying the observer budget *K* and infection rates $\beta$. We are unaware of such an investigation in the literature and believe that it could be of great practical value.

## Results

We conduct our experiments on two synthetic network models—Barabási–Albert^45^ (BA) and Erdős–Rényi^46^ (ER)—as well as on three real world networks. First one is the University of California Irvine ($N=1899,~\langle k \rangle \approx 63.0$, UCI), which contains messages sent among the users of an online community of students from the University of California, Irvine in the U.S.^47,48^. Note that we follow the convention from Spinelli et al.^49^ and Paluch et al.^11^ and modify this graph such that it becomes undirected, without nodes with degree $k < 2$ resulting in $N=1022$ and $\langle k \rangle \approx 12.2$. The second one is the University of Rovira i Virgili ($N=1133,~\langle k \rangle \approx 9.6$, URV) containing email communications at the University Rovira i Virgili in Catalonia in Spain^47,50^. The third one is Infectious—a network built from human face-to-face interactions during the exhibition INFECTIOUS: STAY AWAY in 2009 at the Science Gallery in Dublin^47,51^ ($N=410$, INF). Edges represent contacts which lasted for at least 20 s. Only the data from the day with the most interactions was used. The network is characterised by highest average degree ($\langle k \rangle \approx 84.4$) among all tested graphs.

Three values of infection rate $\beta$ are defined: high ($\beta =0.8$), medium ($\beta =0.5$), and low ($\beta =0.2$)—for a more detailed consideration of infection rates see Supplementary Figs. S27–S28. Four observer placement strategies are evaluated—Betweenness Centrality (BC)^52^ that places observers at the nodes with the highest betweenness centrality, High Coverage Rate (HCR)^10^ that maximises the number of neighbourhoods to which the observers belong, High Variance Observers (HVO)^49^ which maximises the cardinality of the set of nodes lying on the shortest paths between observers, and simply random (RND) as a null model. For location methods, we chose the methods introduced by Pinto et al. (LPTV)^1^ that maximises likelihood estimator using a breadth-first search tree approximation, by Shen et al. (TRBS)^2^ that uses a so-called backwards spreading from observers onto the graph, by Xu et al. (PC)^5^ that relies on the correlation between the topological distance and time of propagation from the source, by Paluch et al. (GMLA)^3^ that modifies the LPTV by introducing a special node selection method, and by Gajewski et al. (EPL)^4^ that also modifies the LPTV to account for loops in the graph. Instead of showing the results as the function of the observer budget, *K*, we chose the observer density $d=K/N$ in our plots to easily compare systems of different sizes. Finally, we chose precision and ${{\,\mathrm{CSS}\,}}_{\alpha }$^11^ as our evaluation metrics (see the “Methods” section for details). Since in the tests, we set $\alpha =0.95$, we will abbreviate ${{\,\mathrm{CSS}\,}}_\alpha$ to just CSS.

**Figure 1:**
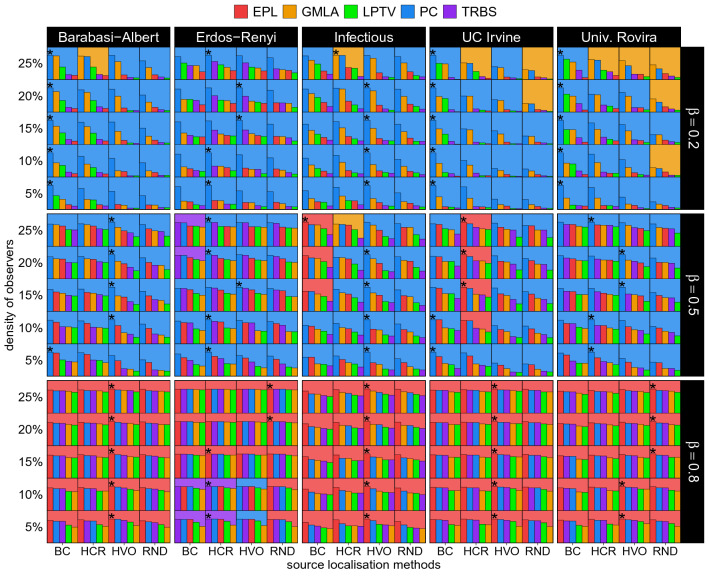
Summary diagrams of precision metric results for all tested networks (major columns) and infection rates $\beta$ (major rows). The colours indicate the localisation methods, whereas observer placement strategies are marked per (minor) column in each block (labels are placed at the very bottom of the plot), while minor rows represent observer densities *d*. Bars within minor blocks show all methods, ordered from the best to the worst (a high precision indicates a high performance), with the background colour of the minor block indicating the best localisation method. The asterisk indicates the best localisation and placement strategy combination per row within a major block, i.e., for a given density, topology and infection rate. Bars are normalised to the highest score per graph, infection rate, and density.

**Figure 2:**
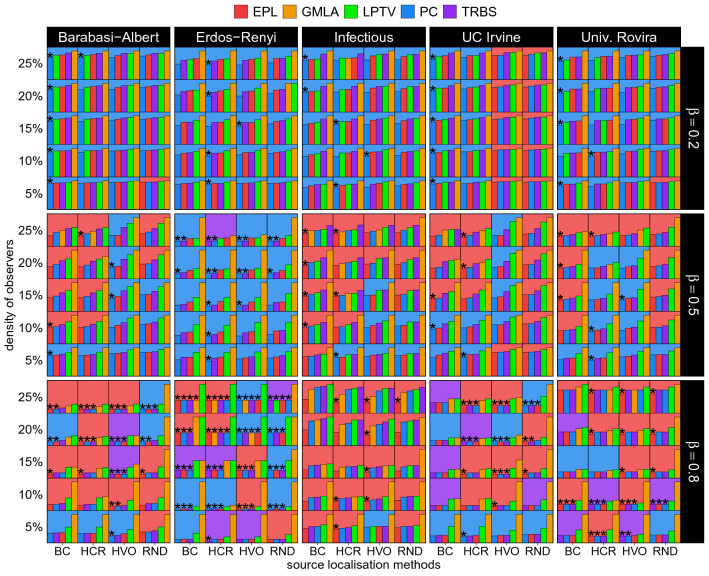
Summary diagrams of CSS metric for all tested networks (major columns) and infection rates $\beta$ (major rows). The colours indicate the localisation methods, whereas observer placement strategies are marked per (minor) column in each block (labels are placed at the very bottom of the plot), while minor rows represent observer densities *d*. Bars within minor blocks show all methods, ordered from the best to the worst (a low CSS value indicates a high performance), with the background colour of the minor block indicating the best localisation method. The asterisk indicates the best localisation and placement strategy combination per row within a major block, i.e., for a given density and topology. Bars are normalised to the highest score per graph, infection rate and density.

Figures 1 and  2 present the main result of our work — a handy summary of all the experiments we have conducted. The diagrams present precision and CSS scores, respectively. Major columns represent the networks (Barabási–Albert, Erdős–Rényi, Infectious, University of Rovira i Virgili, University of California Irvine) while major rows correspond to the different infection rates. Each major block presents performance scores for various densities of observers *d* for every combination of a localisation and placement strategies. The relevant observer placement strategies are indicated in minor rows (the strategies’ labels are listed at the very bottom of the plot) while the relevant localisation methods are indicated by a colour (the colours’ mapping to methods is shown at the top of the diagram). Within each minor block (a tile) we show a bar plot of all the localisation methods, ordered from the best to the worst (the highest to the lowest for precision with the order reversed for CSS). The bar sizes are normalised in respect to the highest score per row, per major block, i.e., per network, infection rate and observer density. The asterisks indicate the best combination of observer placement strategies and localisation methods per row in a major block (see Supplementary Figs. S1–S10 for detailed plots of both precision and CSS for all the methods).

The diagrams’ structure has several levels of detail. At the lowest level, we can see the colour and knowing that each small tile represents a point in our parameter space we can immediately know which localisation method is the best for a given tile. Going upwards, we can evaluate a row of tiles to find, with the help of the asterisk, which combination of observer placement strategies and localisation method is the best for our budget. We can also compare the values between methods for set density and later analyse how these relations change when the density changes by looking at a whole block for a given infection rate and network. At the top level, on the other hand, we can use the colour again to observe regions of dominance of some methods over the others, giving us a bird’s eye view of the parameter space. Not only are these diagrams useful to analyse the vast amount of results we obtained, but they also allow for a swift determination of a suitable method for a similar problem in the real world. While obviously our coverage of possible graphs or infection rates etc. is not exhaustive, it can be an indicator of where to start the process of method selection. A user of these diagrams may first select a panel with the network type closest to the given graph and the infection rate closest to the desired one. Then, the user can find the row with the density closest to the desired density and follow the asterisk in this row to identify the best method to apply. This is of course a simplified scenario, but it illustrates an intended use of our results.

Analysing the diagrams reveals that the PC and EPL methods dominate the landscape, although the TRBS does win in a few cases, e.g., on an Erdős–Rényi graph when the observer density is low (5–10%) and the infection rate is high. Interestingly, while the GMLA method wins many cases with high densities and low $\beta$’s on the URV dataset, it does not take away any asterisks from the PC. Still, making any strong generalisations is difficult here. E.g., the EPL and PC methods have their own respective regions—the EPL is usually preferred at high infection rates and high densities while the PC mostly thrives in complementary cases of a low infection rate and small budget. Observer placement strategy pairing is also quite varied and it does matter what we optimise for—precision or CSS. For instance, on a BA graph, the EPL works better with the HVO in terms of precision, but when the CSS metric is used, the BC is a better choice. The PC most often seems to pair with BC. Yet again, it is not a hard rule. More often than not, it is better to use any placement strategy instead of using randomly placed observers, but even this has a significant number of exceptions. E.g., testing methods on the URV network with precision as metric, the EPL with the random placement strategy wins a high infection rate scenario in four out of five densities. Similarly, for the two highest densities on an ER graph in terms of precision, the EPL pairs best with random placement strategy. It is also apparent that the graph structure affects the best placement strategy. For instance, when optimising for precision on a BA graph, HVO and BC are usually the best while in an ER scenario, the HCR strategy dominates. It is important to recognise that due to the complexity of source localisation, often there is no single best answer. When choosing an appropriate localisation and placement strategies, one must carefully assess the many features of the system.

The rest of this section is dedicated to a more detailed look at the particulars of the experiments we have conducted. Looking at the results for the Barabási–Albert model ($N=1000,~\langle k \rangle =8)$, we can see that for low and medium infection rates, the PC method is most successful. However, for high infection rate, the EPL takes over. In terms of observer placement strategies, when $\beta$ is low, the random placement strategy is definitely the least effective, while the BC is slightly better than the remaining others. For medium and highly infectious regions, the HVO takes the lead. Interestingly, the trends suggest that the GMLA using the BC or HCR placement strategies with high observer budget would win over the PC.

Regarding the CSS metric (for which a low value indicates a high performance) and for low values of infection rate, the PC method dominates again. However, for medium and high values, the PC is in a close competition with the EPL. Similarly to the precision results, the random placement strategy gets the worst results, while other strategies perform closely to each other when $\beta$ is large. For medium and low $\beta$, the BC and HCR placement strategies take the lead.

Examining results for Erdős–Rényi networks, we see a bit different behaviour. The PC method is the most successful across the board with EPL being only ever so slightly lower, but for the high infection rate, the EPL takes over the PC. However, the previously observed GMLA trend is practically absent. Random placement strategy performance is clearly behind the others that perform closely to each other. These outcomes are observed for both metrics, precision and CSS.

We notice that rather peculiar values of the CSS metric may arise for the GMLA method. This method uses a localised gradient-like search for potential sources. Hence, it occasionally fails to even calculate likelihood for the true source. In such a case, all nodes get a maximum ranking equal to the size of the whole graph, resulting in this peculiar value that no other method delivers.

The results on the synthetic networks favour the PC method in most cases. This method also happens to be one of the fastest among those that we test in this review. Table 1 summarises the theoretical and experimental complexities of each method with respect to the system size. It also includes comments regarding the practical considerations. While the tested methods do not seem to differ much in theoretical complexity, their speeds differ in experimental testing—see Supplementary Figs. S11–S12, where we show execution time measurements as a function of both densities and system sizes.

The PC and TRBS are usually the fastest, although the GMLA catches up with them for high observer densities or on large graphs, and can become the fastest. The LPTV and EPL are the slowest. However, for small observer densities, the LPTV is much faster than EPL. For high densities, the EPL does not slow down much while the LPTV slows dramatically so much that it can even become slower than the EPL for some graphs. Similarly, for large graphs, the LPTV could become slower than the EPL because the former’s experimental complexity is slightly higher. While we focused here on synthetic graph with $\langle k \rangle = 8$, the conclusions for both BA and ER hold for other values of $\langle k \rangle$ as well—see Supplementary Figs. S15–S26.

**Table 1:** Computational complexity of the tested methods. We estimated the listed experimental complexities for this review. However, the values of coefficients may change for networks of different sizes or when different hardware or software is used for test execution. For the details on theoretical complexities derivations as functions of observer density, and system size, see the Supplementary Information Sec. Computation time.

Method	Theoretical complexity	Experimental fit $\ln (time) = a \ln (size)+b$	Comments
GMLA	$O(N^{3/2}\log {N})$	$a=1.24(5), b=-7.0(6)\sim N^{1.2}$	Much faster than the EPL and scales better than the LPTV, yet, slower than the PC and the TRBS at low densities or small graphs. At high densities or very large graphs, it is the fastest of all the methods
LPTV	$O(N^2)$	$a=3.20(24), b=-16.3(2.0) \sim N^{3.2}$	In our tests, the complexity turned out to be higher than declared due to the matrix inversion operation. One of the slowest methods
EPL	$O(N^2)$	$a=2.17(11), b=-7.99(97) \sim N^{2.2}$	In our tests, it was the slowest of all the methods due to the very high constant factor (initial cost). However, for high densities or very large graphs, it can actually be faster than the LPTV. Appropriate pre-computing is also possible to mitigate the costs
TRBS	$O(N^2)$	$a=2.17(12), b=-10.4(1.2) \sim N^{2.2}$	One of the faster methods, alongside the PC, except for high density or large scale graphs
PC	$O(N^2)$	$a=2.17(12), b=-10.4(1.2) \sim N^{2.2}$	One of the faster methods, alongside the TRBS, except for high density or large scale graphs

Below, we will analyse the performance of search methods on real world networks. Examining the precision on the University of Rovira i Virgili network, once more, for low and medium infection rates, the PC method dominates, while for high rates, the EPL tops the ranks. The GMLA outperforms the PC method at low $\beta$ when both use the HCR or random placement strategy. However, the PC method combined with BC source localisation method wins decisively in this infection rate region. For medium $\beta$, the HVO placement strategy dominates for high observer densities. Finally, for the high infection rate, random placement strategy performs best with the EPL.

When the CSS metric is used, the outcomes are very similar to those seen for synthetic networks; the PC dominates in low $\beta$, while the PC and the EPL jointly (with few exceptions) take the lead and for this and other cases, the BC placement strategy is the best. We also observe an over-saturation effect when running the PC with the HVO placement strategy for low infection rate (see Supplementary Fig. S3 for the precision curve for this pair of methods). It exhibits a maximum for the value of observer density around $d=0.2$. Such saturation itself is a somewhat expected behaviour since with *N* tending to infinity, the contributions of each subsequent observer are diminishing. However here, we see that these subsequent observers can in fact drastically impede the performance.

The precision results for the University of California Irvine network are distinctly different from the results for other graphs. The most significant is the big difference between performances of the placement strategies. The BC strongly dominates in combination with the PC for small $\beta$. While previously PC dominance continued to medium $\beta$, here actually the EPL combined with the HCR observer placement strategy outperforms the PC. The EPL continues to lead to into high infection rates using the HVO or random positioning placement strategies.

When the CSS metric is used, the outcomes are very similar to those with precision metric. Some differences arise for the very low observer densities, where the PC is slightly ahead of the EPL, but in the medium infection rate range when observer densities exceed 0.1, the EPL outperforms the PC.

The Infectious data set exhibits qualitatively similar behaviour to the other real world graphs in the sense that PC and EPL dominate over other methods, with occasional appearance of GMLA taking the lead with regards to precision. An interesting difference in comparison with UCI and URV is the rather consistent advantage of the HVO placement method, even in low infection rate realm where BC would be the usual winner. This suggests that while location methods have clear leaders (PC and EPL), as far as observer placement is concerned, a more detailed and graph specific approach is recommended.

## Discussion

The main objective for the paper is to provide a comprehensive quantitative comparison of methods for locating an unknown source of spreading process in a known network based on sparse observational data. We have done so by comparing accuracy of several chosen methods in locating the source of a simulated Susceptible-Infected process in synthetic and real networks. The results demonstrate that the methods significantly differ in accuracy and execution times. The method effectiveness depends on such factors as network topology, density of observers, method used to place observers and even specific measure of accuracy used.

While there is no clear single winner among the methods, the study shows that two methods stand above others: a simple Pearson Correlation (PC) and an Equiprobable Links (EPL), with the PC being best for noisy processes with low infection rate $\beta$ and the EPL being best for relatively predictable—yet rapid, aggressive—processes with high $\beta$. While the comparison is interesting in itself, it also allows a more informed choice of methods to use in practical applications. It may be especially interesting to understand why these two methods yielded best results.

The PC method relies on a simple correlation between distance from source and the time it takes for infection to spread. It offers surprisingly high accuracy given how few assumptions and calculations it makes. Unlike methods that incorporate variance (LPTV, GMLA, EPL), it makes no assumptions regarding the distribution of the time it takes to propagate infection along a link. Still, the method only assumes that the mean time should be proportional to distance. It means its assumptions fit well a wide range of scenarios. It is somewhat similar to the TRBS, in that it relies on distance from source, but unlike the TRBS, the former does not actually assume what the mean delay should be, which may be a reason why it handles situations with high uncertainties, such as few observers and low infection rate, better than the TRBS or any other method.

The EPL method is the most complex of the investigated methods and explicitly considers the existence of parallel paths. It assumes the normal distribution to calculate expected variances, which is not consistent with actual delay distributions of Susceptible-Infected processes that have geometric distribution. Multiple parallel paths affect not only variances and covariances of the expected times to arrive at observers starting from a specific potential source, but also their mean values^4^. While the distribution assumptions are mismatched, the inclusion of parallel paths allows this method to best predict the actual characteristics of the spreading process and pinpoint the source with the best accuracy. However, the performance weakens when the spreading process is highly random. This is because the mismatch between natures of assumed and actual distributions increases. A large mismatch lowers the performance. This seems to suggest that the key to the accuracy of these two methods is their ability to cope with full network structure, either by making assumptions simple and general enough to encompass non-treelike structure, or explicitly using that structure for its prediction. This means that the inclusion of the full network structure may be key in development of even more accurate methods, even if it is mathematically challenging. Our work also shows that the choice of the observer placement strategy may play a significant role in overall effectiveness of the search for the source. While the choice does not seem crucial for simple random graphs, the differences in more complex networks is significant—choosing observers based on Betweenness Centrality may improve precision of the PC method over a random placement strategy even by a factor of two, but on the other hand it may actually decrease precision of the EPL method.

The paper describes the current state of the art, but much is yet unknown. As clearly seen from our results, the network structure can heavily affect which localisation method and observer placement strategy work the best together. The nature of the spreading process itself and the observer budget are very significant as well. From our experiments, we can suspect that network features such as degree distribution, clustering coefficient or mean degree have a great impact on the efficacy of the tested methods. Observer placement strategies differ strongly between a scale-free and random graph (degree distribution effect). Similarly, such a difference between the real world graphs is difficult to ignore as well. The choice of the performance metrics is also important as one method can be the best in terms of precision but not in terms of CSS, and vice versa. It is something that should be remembered when determining which approaches to use our results in real world applications.

We hope that the comprehensive results we presented in this paper will serve readers well in both choosing the right methods when building practical applications and focusing on the right topics of further research.

## Methods

### Spreading process

We use an agent-based version of the Susceptible-Infected model^43^ to simulate the spreading process across the system. Each agent in this model may be in one of two states: susceptible (S) or infected (I). Since we conduct our experiments on graphs, each vertex (node) represents an agent and each edge (link) is a part of a possible spreading path. In the beginning of the simulation, we chose one vertex at random and set its status to infected. This node is the true source that the tested methods will attempt to locate. At each time step, the infected agents interact with their neighbours, such that every susceptible neighbour can become infected with a probability $\beta$—the so-called infection rate. To simplify the tracking of propagation paths and infection times, the dynamics of our model is synchronous, which means that at every time step, all infected nodes try to pass the infection simultaneously to their all susceptible neighbours.

This is in contrast, for example, to a normal distribution approach used by Pinto et al.^1^ that can generate certain awkwardness we would like to avoid. It is potentially possible to cause a “time travel” scenario when the weights drawn are negative, thus requiring the normal distribution to be appropriately truncated^53^. Such truncation naturally changes the moments of the distribution and thus introduces errors to our estimators. Additionally, the normal distribution does not directly model any common spreading phenomena such as epidemics or fake news cascades. In this paper, we study the effectiveness of source localisation using the SI model even if it is in disagreement with assumptions required by the tested methods.

### Evaluation metrics

We use two efficiency measures in this paper: the average precision and the Credible Set Size.

#### Precision

The precision for a single test is defined as the ratio between the number of correctly located sources (i.e., true positives, which here equals either zero or one) and the number of sources found by the method (i.e., true positives plus false positives, which here is at least one). The tests are repeated multiple times for different origins, many graph realisations (for synthetic networks) and then the obtained values of precision are averaged.

#### Credible Set Size

The Credible Set Size at a chosen confidence level of $\alpha$ (${{\,\mathrm{CSS}\,}}_{\alpha }$) is a novel metric introduced by Paluch et al.^11^. It represents the size of the smallest set of nodes containing the true source with the probability $\alpha$. In other words, it allows us to determine how many nodes with the highest *score* must be labelled as the origin of the spread to reach probability $\alpha$ of the true origin being among these nodes. We compute this probability as the hit rate (recall) from many realisations of signal propagation and source localisation.

### Observer placement strategies

#### Betweenness centrality: BC

It is a fairly simple but effective heuristic that selects those nodes that maximise a measure called *betweenness centrality*^52^, computed independently for every node $v \in V$ as

1 $$\begin{aligned} S_{\text {BC}} = \mathop {\text {argmax}}\limits _{S} \sum _{v \in S} \sum _{\begin{array}{l} i,j \in V \\ i\ne j \ne v \end{array}} \sigma _{ij}^{(v)}/\sigma _{ij}, \end{aligned}$$

where $\sigma _{ij}^{(v)}$ is the number of shortest paths between nodes *i* and *j* which contain *v* and $\sigma _{ij}$ is the total number of shortest paths between these nodes.

#### High coverage rate: HCR

This method^10^ selects such a set of observers $S_\text {HCR}$ that maximises the number of unique neighbours of observers. Neighbours of observers are labelled as *covered*, and the fraction of such covered nodes in the network is called the *coverage rate* $C_S$ and formally defined as:

2 $$\begin{aligned} C_S = \frac{\left| \bigcup _{i=1}^K T_i\right| }{N}, \end{aligned}$$

where *K* is the number of observers, $T_i$ is the neighbour set of the observer $o_i$ and *N* is the size of the network. We seek such a set $S_{HCR}$ that maximises $C_S$.

This method quite naturally saturates, i.e., it is possible to get coverage rate of one while the density of observers is smaller than needed for testing. Such saturation is desired in real world applications, but it can pose a problem when comparing this method with others. To avoid this problem, we extend this approach such that the observers are chosen greedily, one by one, and each new observer increases the coverage rate until it reaches unity. Then we select observers maximising the number of nodes which have two observers as their neighbours (*double-covered* nodes). We continue similarly to find the maximum number of *triple-covered* nodes and so on until the desired density of observers is reached.

#### High variance observers: HVO

This method^49^ uses a path covering strategy. One seeks such a set of observers $S_{\text {HV-Obs}(L)}$ that maximises the cardinality of the set of nodes—$P_L(S)$—lying on the shortest paths of length at most *L* between any two observers in the set *S*.

This approach performs best when the density of observers is small since then $P_L(S)$ increases quickly and can easily include all nodes in the network. We extend this method as well similarly to the case of the High Coverage Rate method. A node which lies on an exactly one shortest path of length at most *L* between any two observers in the set *S* is called the *single-path-covered* while a *double-path-covered* node lies on two shortest paths, *triple-path-covered* lies on three shortest paths and so on. We select greedily the observers maximising the number of *single-path-covered* nodes until all nodes are *single-path-covered*. Then the number of *double-path-covered* is maximised and we continue in this manner until the desirable density of observers is reached.

#### Random: RND

We consider it a suitable null model for observer placement strategy to simply distribute them randomly in the graph.

### Source localisation

Here, we briefly describe each of the procedures used to estimate the source of a propagation. Each method shown here in essence calculates a *score* for each node *s* denoted as ${\mathscr {F}}(s)$. Then, the estimated source ${\hat{s}}$ of the propagation on a graph ${\mathscr {G}}$ is each node with the highest score, more formally:

3 $$\begin{aligned} {\hat{s}} = \mathop {\text {argmax}}\limits _{s\in {\mathscr {G}}}{\mathscr {F}}(s). \end{aligned}$$

#### Pinto, Thiran, Vetterli method: LPTV

Pinto et al.^1^ introduce a maximum likelihood estimator of the form (3), show it is optimal on trees and that it is quite successfully applicable to general graphs. The method is conceptually similar to locating a cell phone using information about this phone from the telecommunication towers^54^.

Let $|{\mathscr {P}}(u,v)|$ denote the length of the path ${\mathscr {P}}(u,v)$ connecting nodes *u* and *v*, *d* the vector of observed delays, ${\mu }_s$ the vector of deterministic delays, and ${\Lambda }_s$ the delay covariance matrix, then the score is:

4 $$\begin{aligned} {\mathscr {F}}(s) = \mu _{s}^{T} \Lambda ^{-1} (d - \frac{1}{2}\mu _s), \end{aligned}$$

where:

5 $$\begin{aligned} \begin{aligned}{}[{d}]_k&= t_k - t_0, \\ [{\mu }_s]_k&= \mu (|{\mathscr {P}}(s,o_k)|-|{\mathscr {P}}(s,o_0)|),\\ [{\Lambda }_s]_{k,i}&= \sigma ^2 |{\mathscr {P}}(o_0,o_k)\cap {\mathscr {P}}(o_0,o_i)|, \;\; for \;k,i=1,\dots ,K-1. \end{aligned} \end{aligned}$$

In the original paper, the authors use the information about the node that sent the signal to the given observer. We do *not* use this information, as it is not always available in real world scenarios. Therefore from now on, when we refer to the LPTV, we mean a *limited information* variant of it. The same limitation applies to other works cited in this paper.

#### Time reversal backwards spreading: TRBS

Shen et al.^2^ propose a method that conducts so-called backwards spreading. In essence, it finds topologically shortest paths from the observers to a potential source and compares those to observed reception times. More precisely, it constructs a vector *T* where $[T_s]_i = d_i - [\mu _s]_i$ for each observer *i* and node *s*. We are using notation consistent with the one used in (4). However, there is no reference observer needed in this approach, so:

6 $$\begin{aligned} \begin{aligned}{}[{d}]_k&= t_k, \\ [{\mu }_s]_k&= \mu |{\mathscr {P}}(s,o_k)|,\; k = 0, \ldots ,K-1.\\ \end{aligned} \end{aligned}$$

The node that is most likely the source is the one that *minimises* the variance of the vector *T*. To keep the scoring consistent with other methods and Eq. (3) we define the score function as:

7 $$\begin{aligned} {\mathscr {F}}(s) = -Var(d - \mu _s). \end{aligned}$$

#### Pearson correlation: PC

Xu et al.^5^ present two hypothesis (we quote verbatim):

- “The distance between the neighbour node who transmits the epidemic or information to the observation node and the source node should be closer to the distance between the observation node and the source node.”
- “The temporal order of the observation nodes getting infected or informed should be consistent with the length order of the distance between the observation nodes and the source nodes.”

The authors use Pearson correlation coefficient as the measure of the correlation between the topology of the network and the infection times. Thus, the score definition in this case is:

8 $$\begin{aligned} {\mathscr {F}}(s) = \frac{\sum _{i=0}^{K-1}([\mu _s]_i - \overline{\mu _s})(d_i - {\overline{d}}) }{ \sqrt{ \sum _{i=0}^{K-1} ([\mu _s]_i - \overline{\mu _s})^2 \sum _{i=0}^{K-1}(d_i - {\overline{d}})^2 } }. \end{aligned}$$

#### Gradient maximum likelihood algorithm: GMLA

A modification of LPTV presented by Paluch et al.^3^ introduces two improvements: a limited number of observers, and a gradient-like selection of suspected nodes. The first is motivated by realisation that observers that are very far from the spread source make a very small contribution to the score in comparison to the nearest observers, while those distant observers greatly increase the cost of information processing. It is very likely that the source of the spread is in close proximity to the observer with the smallest recorded time (the observer one) and so the method only uses $K_0$ observers with smallest recorded times for calculating scores. The procedure starts by calculating the scores of the nearest neighbours of the observer one and then selects a neighbour with the highest score. Next, the method goes forwards from this node and calculates scores for its nearest neighbours looking for a node with a score greater or equal to the current maximum. This process continues until all neighbours have a score lower than the current maximum.

#### Equiprobable links: EPL

As shown by Gajewski et al.^4^ the assumption of a tree-like propagation is not always satisfied. Treating paths as random variables makes the actual expected time of traversal equal to the first moment of a minimum distribution of all paths connecting two nodes. Similarly, covariances between observers become covariances of multivariate minimum distribution. Since establishing general expression for such minimum distribution of an ensemble of *n* paths is a non-trivial task^55^, even with an *identically and independently distributed* random variables approximation^4^, one can settle for a known analytical formula in the case of $n=2$ discussed by Nadarajah and Kotz^56^:

Let $Y = \min (X_1, X_2)$ where $X_1, X_2$ are known Gaussian distributions such that $E[X_i] = \mu _i, E[X_i^2]-E[X_i]^2 = \sigma _i^2$, and $\Phi , \phi$ are respectively the cumulative distribution function and pdf of the standard normal distribution. Thus

9 $$\begin{aligned}&E[Y] = \mu _1\Phi \big (\frac{\mu _2 - \mu _1}{\theta } \big ) + \mu _2\Phi \big (\frac{\mu _1 - \mu _2}{\theta } \big ) - \theta \phi \big (\frac{\mu _2 - \mu _1}{\theta } \big ), \end{aligned}$$

10 $$\begin{aligned}&E[Y^2] = (\sigma _1^2 + \mu _1^2)\Phi \big (\frac{\mu _2 - \mu _1}{\theta } \big ) + (\sigma _2^2 + \mu _2^2)\Phi \big (\frac{\mu _1 - \mu _2}{\theta } \big ) - (\mu _1 + \mu _2)\theta \phi \big (\frac{\mu _2 - \mu _1}{\theta } \big ),~\text {with}~ \end{aligned}$$

11 $$\theta = \sqrt{\sigma _1^2 + \sigma _2^2 - 2\rho \sigma _1\sigma _2},$$

where $\rho$ is a correlation coefficient between the distributions $X_1, X_2$ that in case of paths we define as number of common edges between the paths ${\mathscr {P}}_1, {\mathscr {P}}_2$ normalised by the length *L* of the path:

12 $$\rho = \frac{{\mathscr {P}}_1 \cap {\mathscr {P}}_2}{L}.$$

Using the above equations, we can compute the deterministic vector $\mu _s$ as well as the covariance matrix $\Lambda$ as defined by Eq. (4). While the former is quite straightforward, the latter is not. We get $\mu _s$ by treating each shortest path *i* as a random normally distributed variable with mean $L \mu$, variance $L \sigma ^2$ correlated with another path *j* - $\rho (i, j)$. Since we are using a two-path approximation described above, for $n>2$ paths, we assume there are exactly two paths with $\rho = 0$ and mean defined by Eq. (9).

To compute the covariacne matrix $\Lambda$ that we use in the EPL, we apply the same score function as in the LPTV defined by Eq. (4).

Let ${\mathscr {E}}_i$ represent a set of paths connecting node $o_i$ to the reference observer $o_0$:

13 $${\mathscr {E}}_i := \{{\mathscr {P}}_1(o_{0}, o_{i}), {\mathscr {P}}_2(o_{0}, o_{i}), \dots , {\mathscr {P}}_n(o_{0}, o_{i})\},$$

where ${\mathscr {P}}_i(x, y)$ is an $i-th$ path between vertices *x* and *y*. Using Eq. (4), we define the covariance matrix in which each element is a measure of the correlation between the sets of paths:

14 $$\begin{aligned} {\Lambda }_{i,j} &= \frac{|\{e_i\}\cap \{e_j\}|}{|\{e_i\}\cup \{e_j\}|}\cdot \sqrt{\min _{\sigma ^2}({\mathscr {E}}_{i})\cdot \min _{\sigma ^2}({\mathscr {E}}_{j})}, \\& \quad \text {with} \; \; \min _{\sigma ^2}({\mathscr {E}}_{i} ) = E[Y^2] - E[Y]^2,\; \text {using}\; Eq. \; (9), (10), \end{aligned}$$

where $\{e_i\}$ is a set of edges of all (shortest) paths connecting node *i* with the reference observer. A possible interpretation of using an intersection instead of a union is that we consider each edge equally likely to be a part of propagation. Here, of course, we use a measure of correlation known as the Jaccard Index^57^.

## Experimental setting

Simulations on Barabási–Albert and Erdoős–Rényi graphs were conducted on a machine with the AMD®Ryzen 7 1800X while those on University of Rovira i Virgili, and University of California Irvine datasets were run on the Prometheus cluster node made of two Intel Xeon E5-2680 v3 processors^58^ Experiments depicted in Supplementary Figs. S5, S10, S15–S28 (in Supplementary Information) and on the Infectious dataset were performed on an AMD®EPYC 7452 machine.

## Supplementary Information

Supplementary Figures.
