# Supplementary material for: Comparison of observer based methods for source localisation in complex networks
Source: Sci Rep. 2022 Mar 24;12:5079. doi: 10.1038/s41598-022-09031-0 (PMC8948209; doi:10.1038/s41598-022-09031-0)
Supplement: Supplementary file 1 — Supplementary Figures. [file 41598_2022_9031_MOESM1_ESM.pdf]

Source localisation with limited information Supplementary Information

Łukasz G. Gajewski[1,*], Robert Paluch[1], Krzysztof Suchecki[1], Adam Sulik[1], Bolesław K. Szymański[2,*], and Janusz A. Hołyst[1,3]

[1]Center of Excellence for Complex Systems Research, Faculty of Physics, Warsaw University of Technology, Koszykowa 75, 00-662, Warsaw, Poland
[2,*]Network Science and Technology Center, Rensselaer Polytechnic Institute, 110 8th St, Troy, NY 12180, USA
[3]ITMO University, Kronverkskiy Prospekt 49, St Petersburg, Russia 197101
[*]lukaszgajewski@tuta.io

Detailed precision results for synthetic networks

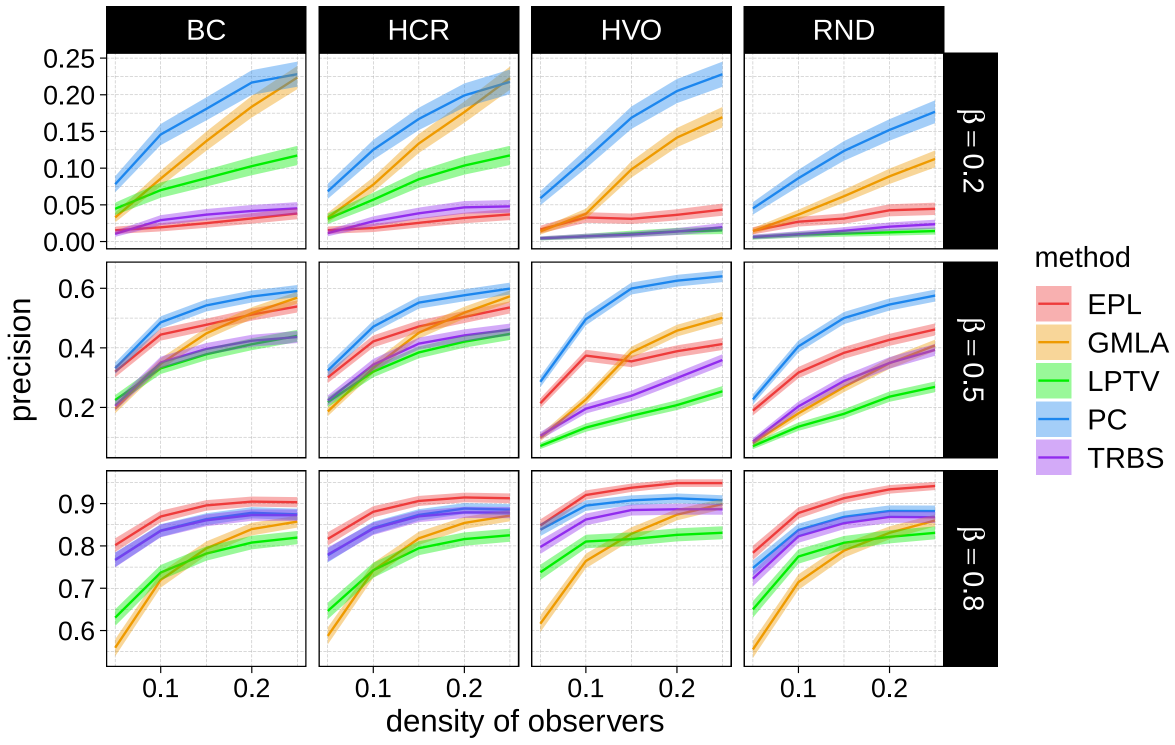

Figure S1. Precision of source localisation on a Barabási-Albert graph ($N = 1000$, $\langle k \rangle = 8$) as a function of observer density, with various observer placements and three different values of infection rate $\beta \in \{0.2, 0.5, 0.8\}$. Each data point is an average of $1.5 \cdot 10^4$ realisations with error bands representing 5 standard deviations.

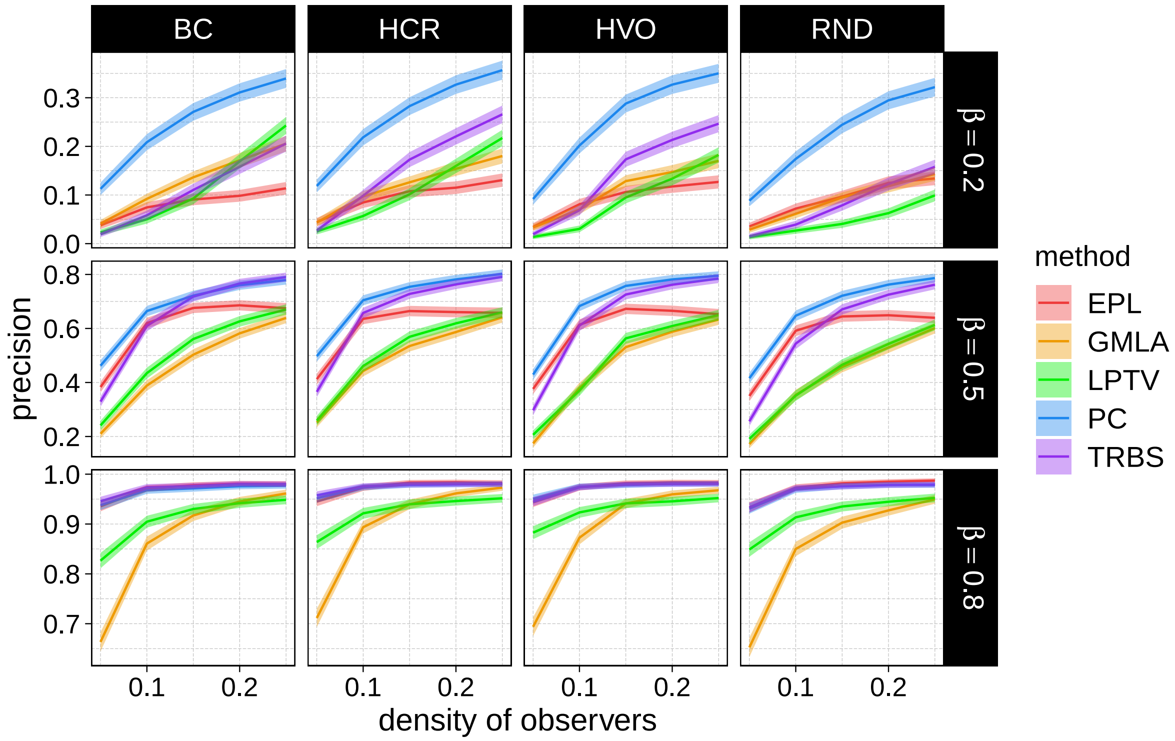

Figure S2. Precision of source localisation on an Erdős-Rényi graph ($N = 1000,\ \langle k \rangle = 8$) as a function of observer density, with various observer placements and three different values of infection rate $\beta \in \{0.2, 0.5, 0.8\}$. Each data point is an average of $1.5 \cdot 10^4$ realisations with error bands representing 5 standard deviations.

Detailed precision results for real-world networks

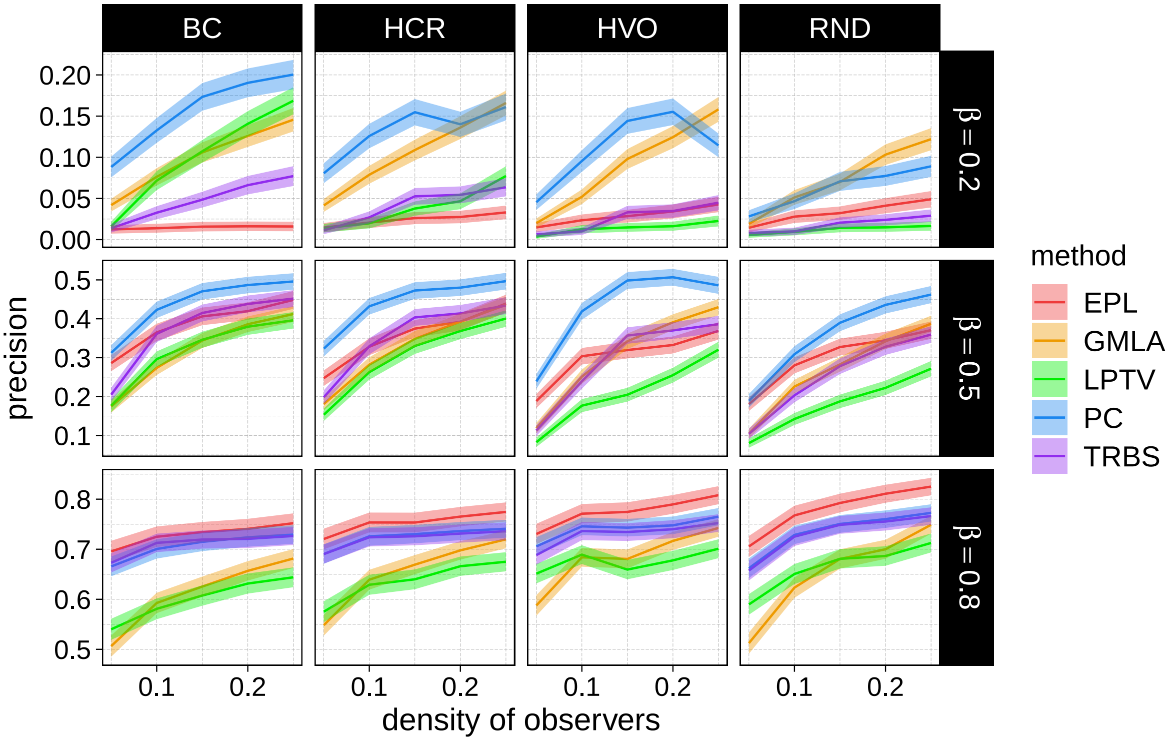

Figure S3. Precision of source localisation on an real world graph - University of Rovira i Virgili - as a function of observer density, with various observer placements and three different values of infection rate $\beta \in \{0.2, 0.5, 0.8\}$. Each data point is an average of $1.1 \cdot 10^4$ realisations with error bands representing 5 standard deviations.

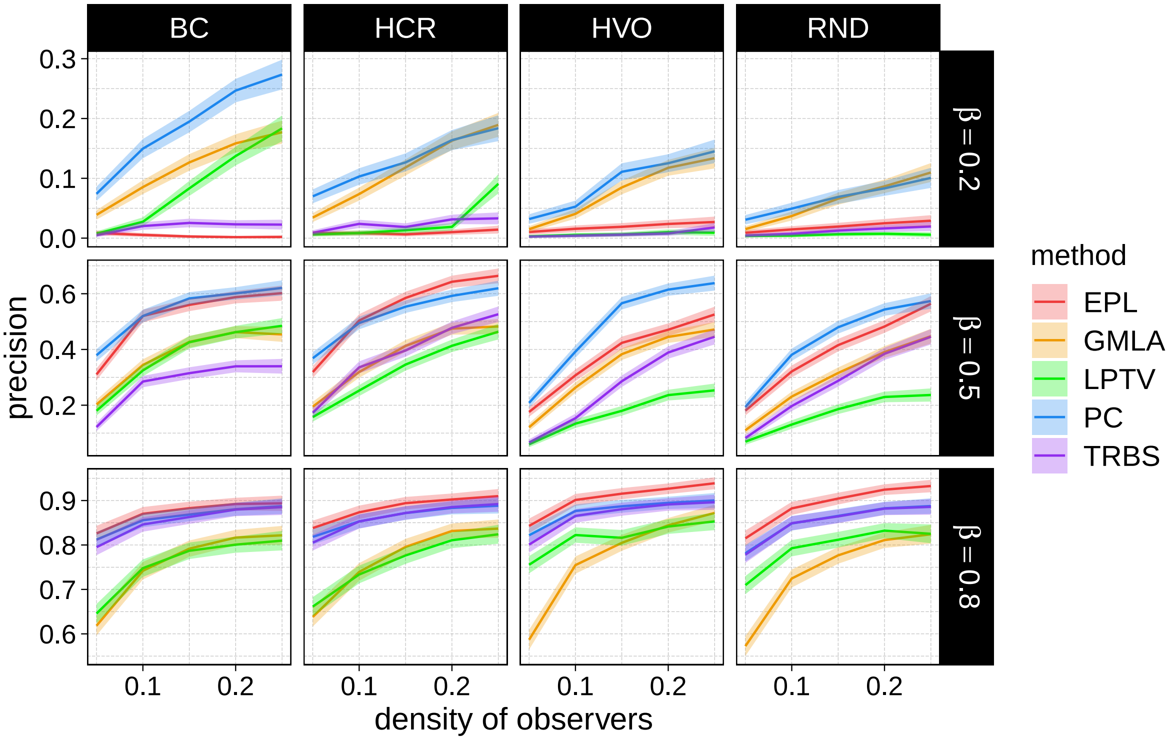

Figure S4. Precision of source localisation on an real world graph - University of California Irvine - as a function of observer density, with various observer placements and three different values of infection rate $\beta \in \{0.2, 0.5, 0.8\}$. Each data point is an average of $1.1 \cdot 10^4$ realisations with error bands representing 5 standard deviations.

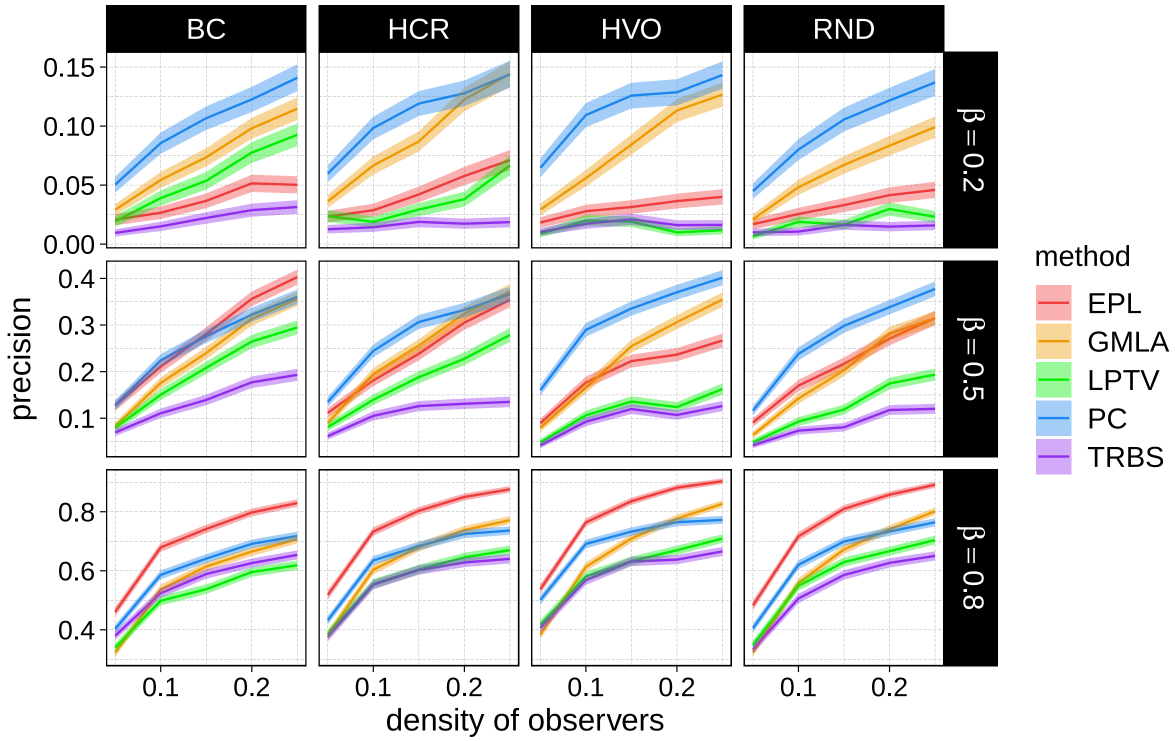

Figure S5. Precision of source localisation on an real world graph – Infectious – as a function of observer density, with various observer placements and three different values of infection rate $\beta \in \{0.2, 0.5, 0.8\}$. Each data point is an average of $2.25 \cdot 10^4$ realisations with error bands representing 5 standard deviations.

Detailed α-CSS results for synthetic networks

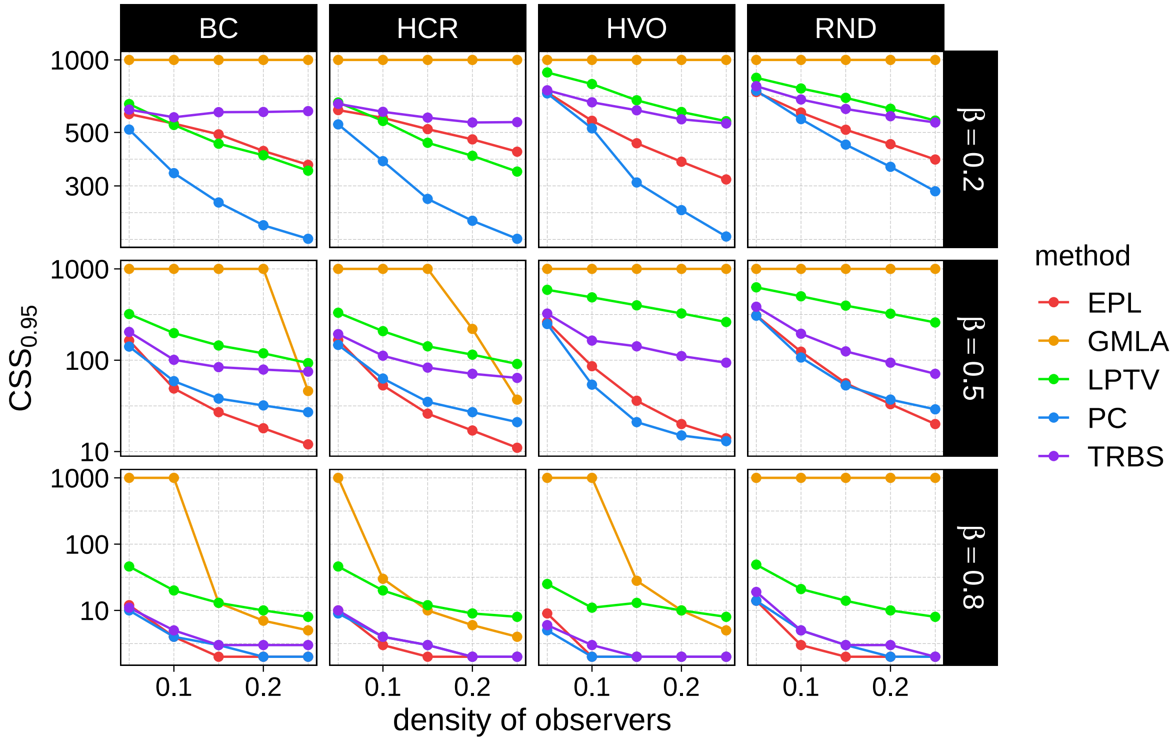

Figure S6. 0.95-CSS of source localisation on a Barabási-Albert graph ($N = 1000$, $\langle k \rangle = 8$) as a function of observer density, with various observer placements and three different values of infection rate $\beta \in \{0.2, 0.5, 0.8\}$, after $1.5 \cdot 10^4$ realisations.

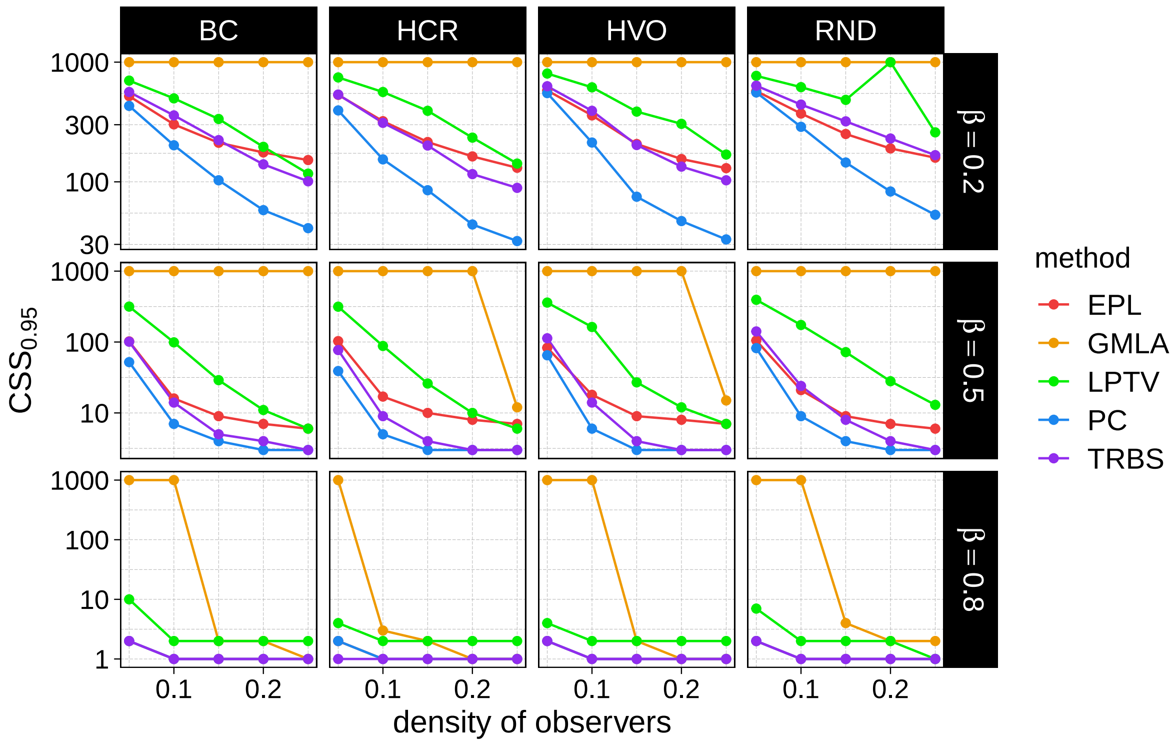

Figure S7. 0.95-CSS of source localisation on an Erdős-Rényi graph ($N = 1000$, $\langle k \rangle = 8$) as a function of observer density, with various observer placements and three different values of infection rate $\beta \in \{0.2, 0.5, 0.8\}$, after $1.5 \cdot 10^4$ realisations.

Detailed α-CSS results for real-world networks

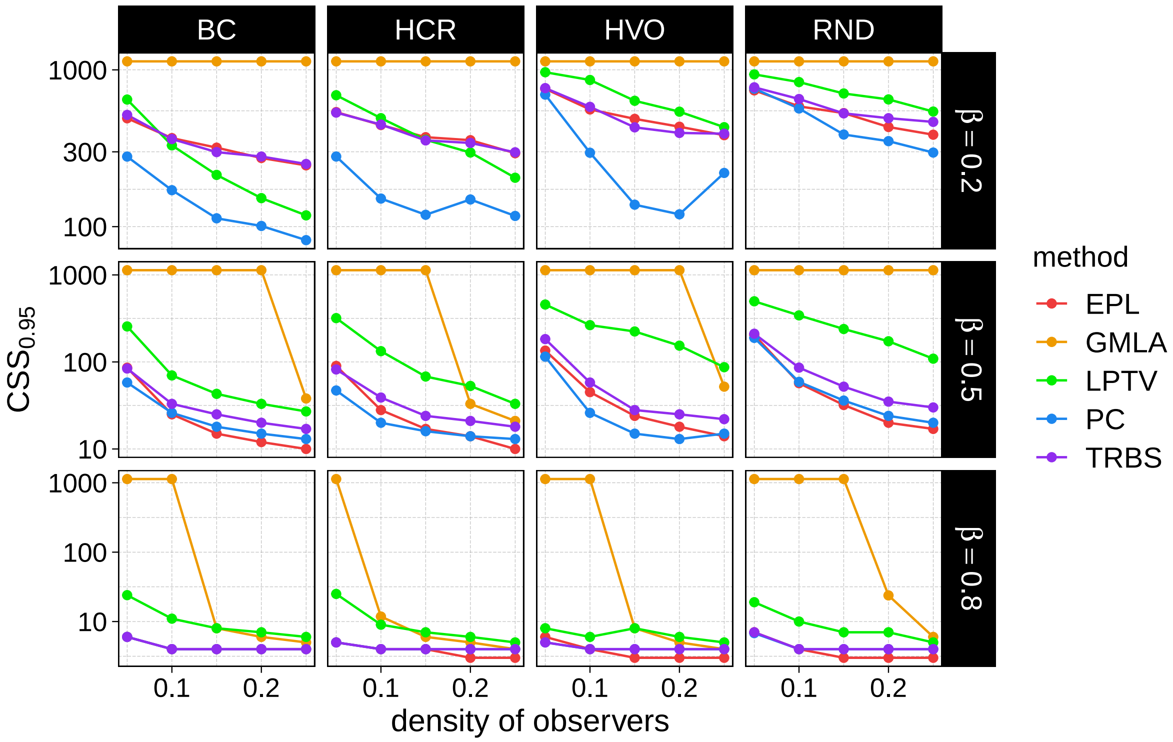

Figure S8. 0.95-CSS of source localisation on an real world graph - University of Rovira i Virgili - as a function of observer density, with various observer placements and three different values of infection rate $\beta \in \{0.2, 0.5, 0.8\}$, after $1.1 \cdot 10^4$ realisations

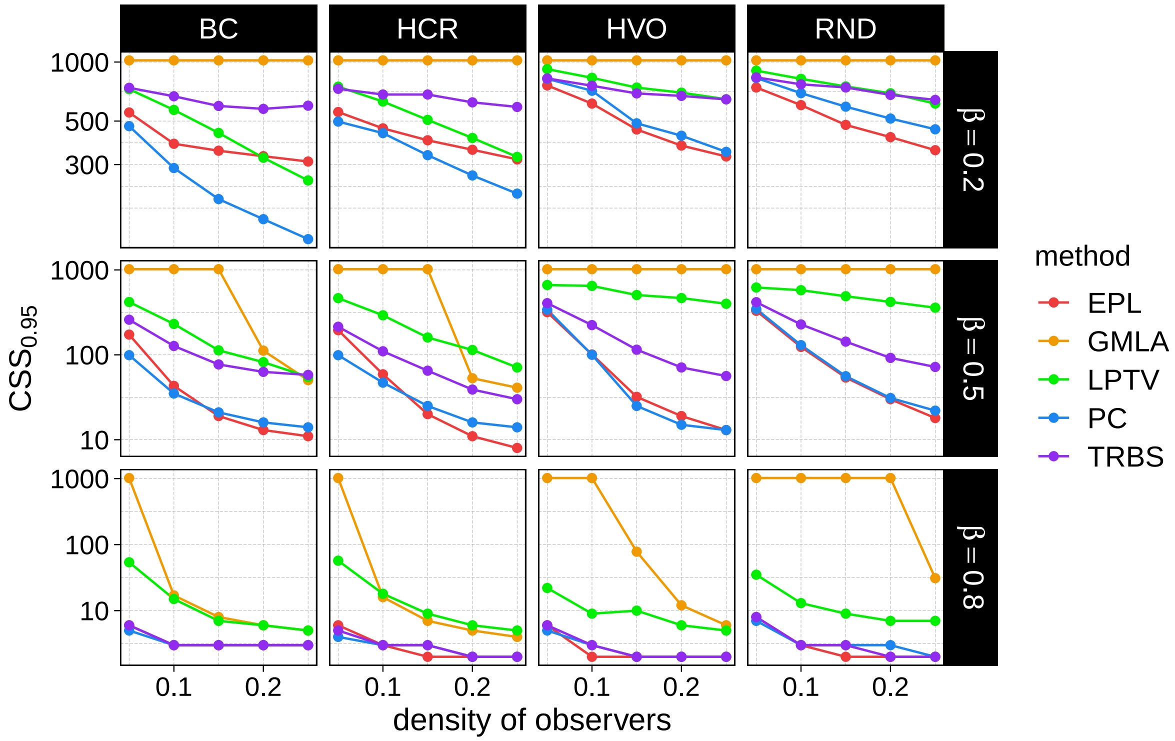

Figure S9. 0.95-CSS of source localisation on an real world graph - University of California Irvine - as a function of observer density, with various observer placements and three different values of infection rate $\beta \in \{0.2, 0.5, 0.8\}$, after $1.1 \cdot 10^4$ realisations

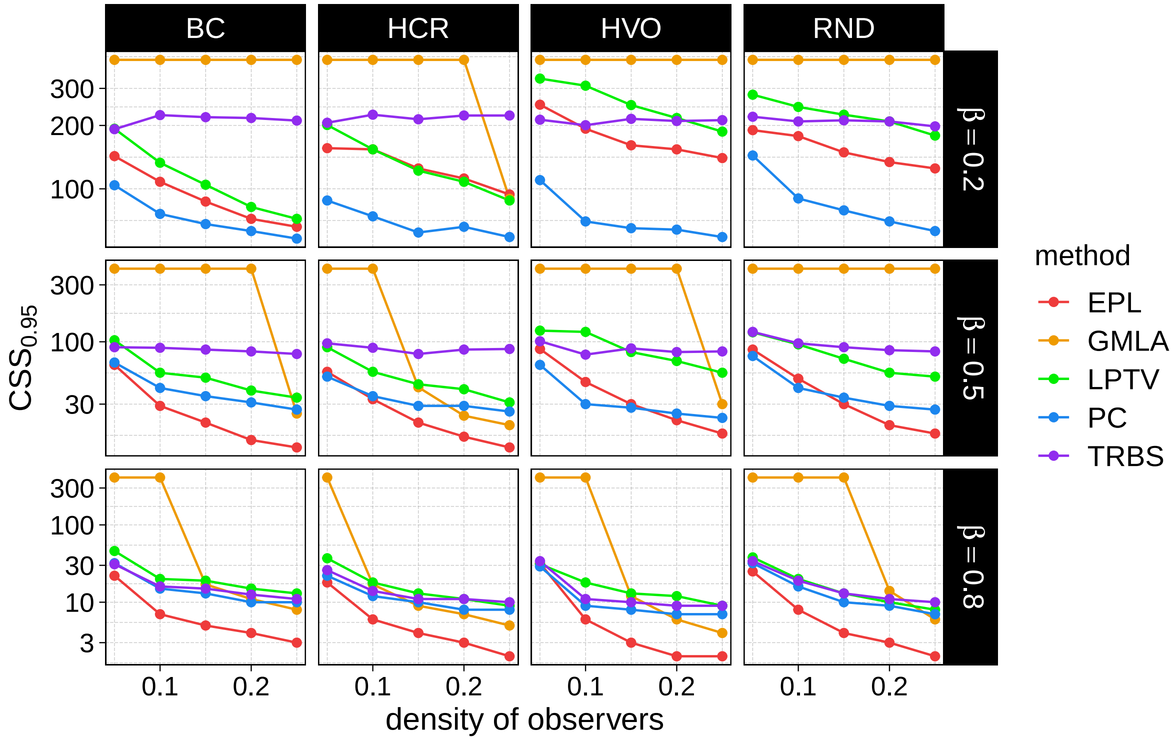

Figure S10. 0.95-CSS of source localisation on an real world graph – Infectious – as a function of observer density, with various observer placements and three different values of infection rate $\beta \in \{0.2, 0.5, 0.8\}$, after $2.25 \cdot 10^4$ realisations

Computation time

Not all the methods used by us have their complexity declared clearly in the original papers and those that do often have slightly different assumptions in their analysis. As such here we try to unify the complexity analysis for all the methods.

Starting with LPTV, in the original paper authors claim $O(N^3)$ assuming that a breadth-first search (BFS) tree is $O(N^2)$ and it is done for every node. However, there are two aspects to be addressed here. Firstly, the complexity of a BFS is $O(E+N)$ (where E is the number of edges) and if the mean degree $\langle k \rangle \to \infty$ as $N \to \infty$ then it is indeed $O(N^2)$. Using an example of a full graph for simplicity, we get $E = N(N-1)/2$ hence $O(N^2+N) = O(N^2)$. On the other hand if $\langle k \rangle \to$ const. as $N \to \infty$ then we have $O(\langle k \rangle N + N) = O(N)$. Secondly, and in this case more importantly, since Pinto et al. assume the *number* of the observers to stay constant, they neglect the complexity of matrix inverse operations. Since we assume the *density* of the observers to be constant we can no longer do that. Matrix inverse complexity varies depending on the method but assuming the Gauss algorithm, for an $n \times n$ matrix we have $O(n^3)$. In our case $n = d \cdot N$ where d is the observer density. As such the matrix inverse becomes $O(N^3)$ and we need to do that for each node hence the complexity of the LPTV method is in fact $O(N^4)$, regardless of how we compute the complexity of the BFS as the matrix inverse becomes the dominant factor.

For the related method - EPL, via a very similar reasoning we get $O(N^3)$. Again the matrix inverse operation is the dominant factor, however, unlike in LPTV we only do it once and not for every node separately.

GMLA, also related to LPTV, being a gradient variant with limited number of observers, takes the order of $\sqrt{N}$ observers (instead of $O(N)$) and computes the score for $\log(N)$ nodes with yet again having the matrix inverse as the bulk of the computation giving $O(N^{3/2}\log(N))$. Note that the $\sqrt{N}$ rule is not set in stone and can be chosen to be different, thus changing the exponent of $N^{3/2}$.

TRBS and PC are very similar to each other as their computation is dominated by finding shortest paths and computing an $O(K)$ measure on a set of K observers (either a variance in case of TRBS or a Pearson's coefficient in PC). As such we get for both of them either $O(N^3)$ if $\langle k \rangle \to \infty$ (see the analysis of LPTV above) or $O(N^2)$ otherwise. Do note that in the their original paper Shen et al. provide $O(N^2 \log(N))$ without much explanation. We suspect this is due to a weighted graph shortest path algorithm complexity (Dijkstra) being conducted for each node giving $N \cdot N \log(N)$. Since here we do not consider a weighted variant of the localisation problem we can use the BFS algorithm instead of Dijkstra.

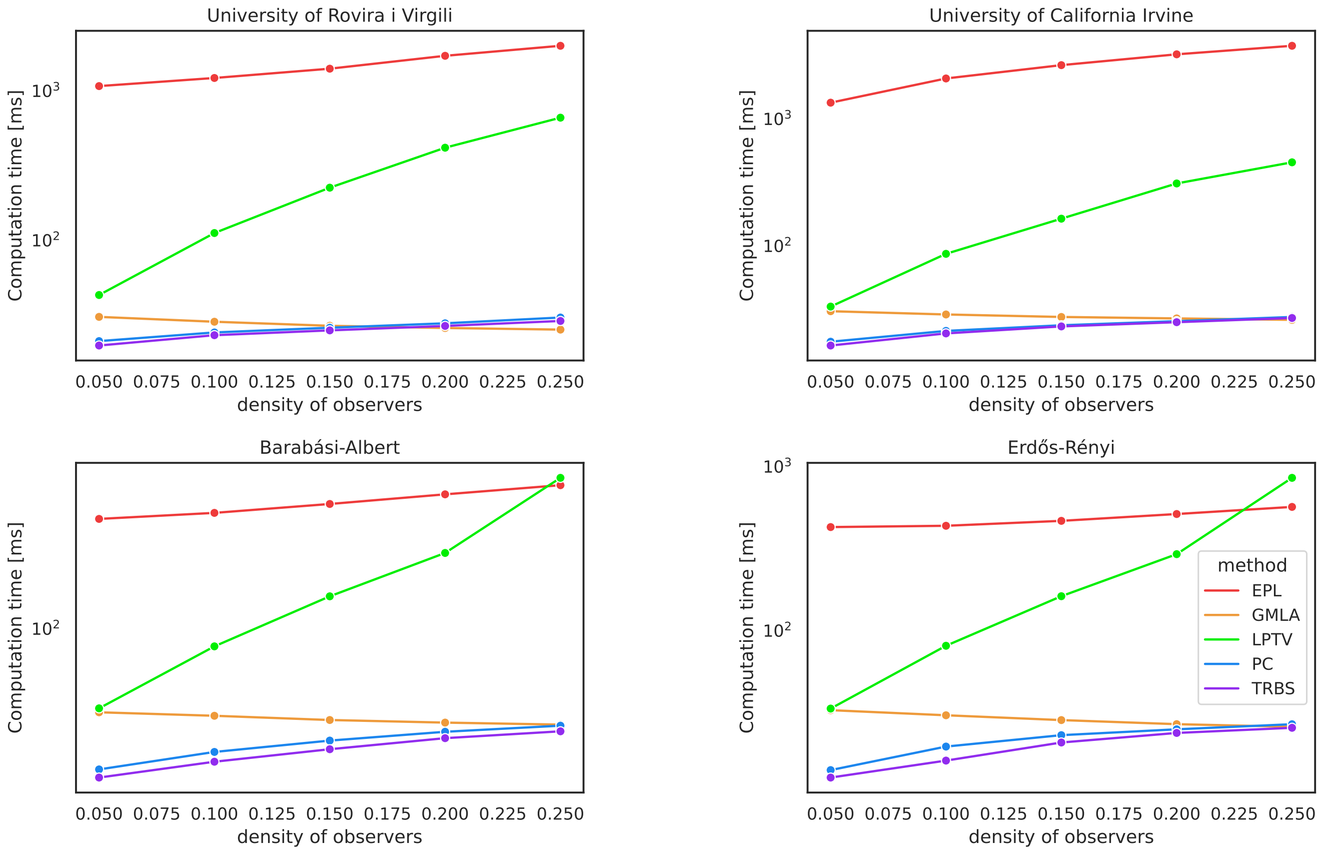

Figure S11. Average recorded computation time amongst all realisations for each tested localisation method as a function of observer density. Panel titles indicate what graph was used for the test and they correspond to the systems used in all other results presented in this paper. Simulations on Barabási-Albert and Erdős-Rényi graphs were conducted on a machine with the AMD® Ryzen 7 1800X while University of Rovira i Virgili, and University of California Irvine on the Prometheus cluster node made of two Intel Xeon E5-2680 v3 processors. We choose the average recorded time since the algorithms (especially GMLA) are nondeterministic and therefore other measures (such as lowest recorded time) would be inadequately favouring GMLA.

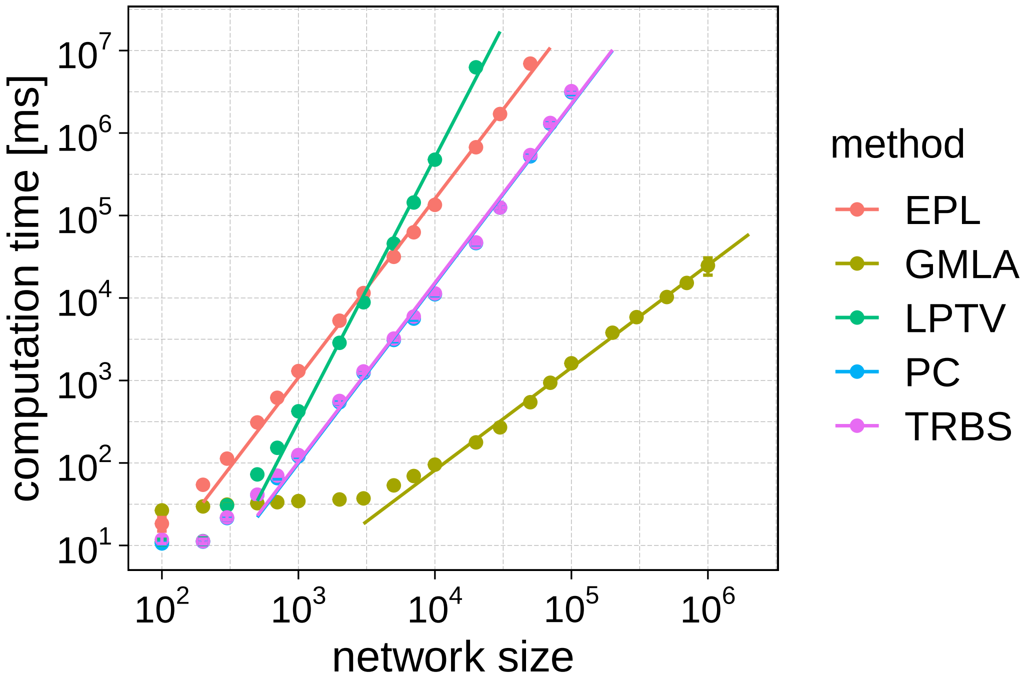

Figure S12. Comparison of the running times of different algorithms. All tests are performed on Erdős-Rényi graph with $\langle k \rangle = 8$. Observers are placed randomly with $\rho = 0.05$. Infection rate $\beta = 0.5$ is used for all simulations. Every point is an average over many (50-12000) realisations. Error bars show 5 times the standard errors. Solid lines are linear models $\ln(time) = a\ln(size) + b$, where $a_{\text{EPL}} = 2.17(11), b_{\text{EPL}} = -7.99(97)$, $a_{\text{GMLA}} = 1.24(5), b_{\text{GMLA}} = -7.0(6)$, $a_{\text{LPTV}} = 3.20(24), b_{\text{LPTV}} = -16.3(2.0)$, $a_{\text{PC}} = 2.17(12), b_{\text{PC}} = -10.4(1.2)$, $a_{\text{TRBS}} = 2.17(12), b_{\text{TRBS}} = -10.4(1.2)$. The fits are made for the networks of sizes of at least 10^3 nodes., except GMLA for which the minimum size of network is 10^4 nodes. The numbers in parenthesis show 95% Confidence Interval. Simulations were conducted on a machine with the AMD® EPYC™ 7452 2.35GHz.

Location performance response to observer density

Intuitively we would expect the precision to be a monotonously non-decreasing function of the observer density, however, amongst our experiments we observer several examples of this intuition being broken. See, e.g., Fig. S1 EPL with HVO for $\beta = 0.5$ a downward trajectory from density $d = 0.1$ to $d = 0.15$ or Fig. S3 PC with HVO for $\beta = 0.2$, densities $d = 0.15$ and $d = 0.2$ (this list is not exhaustive).

In this section we propose three possible explanations for this, although in earnest we are unable to conclusively answer why this happens.

Firstly, it is possible that our statistics are insufficient. Despite conducting tens of thousands of simulations the complexity of the problem can be such that it requires orders of magnitude more to reach something of a stable relations of precision and observer density. To test this we took 70 different realisations of a Barabási-Albert graph and simulated 100 Susceptible-Infected cascades on it, attempted to find the source, and analysed each BA instance separately. All of this data combined is what is presented in main text and in other sections of this supplementary information. In Fig. S13 we show box plots for each of this instances for different infection rates (β) for the aforementioned downward transition for EPL and HVO, i.e., $d = 0.15$. What can be seen is that the results can indeed vary widely for the same *type* even with a relatively high β. Additionally, to exclude the effects of not sufficient statistics, we have used the Kruskal's test for the variant EPL+HVO, $\beta = 0.5$ (see Fig. S1), and obtained significance level $\alpha = 0.025$ that we can reject the hypothesis of the precision for $d = 0.1$ and $d = 0.15$ being equal. Moreover, Welch's Two-sample T-test shows that we can reject this hypothesis on the significance level $\alpha = 0.008482$ in favor of the alternative hypothesis that the precision for $d = 0.1$ is indeed higher than for $d = 0.15$. One can visually inspect this with the Fig. S14 showing box plots of precision as a function of the density for EPL and HVO.

Secondly, HVO is a rather particular placement strategy that the different density level observer set are not necessarily correlated. What we mean here is that usually on a given graph the best observers set for density d_1 are part of the best observers set for $d_2 > d_1$. This is true for all placement methods except for HVO where observer set for two different densities can be completely different, and thus the response in precision can appear non-smooth.

Thirdly, the potential statistical variance effects described above cannot explain the discussed phenomenon in the case of real-world networks. When we use such a network it is set for every SI realisation, and thus no such variance exists. Similarly, the decreasing trend can also appear for other placement methods (see, e.g., Fig. S3, $\beta = 0.2$, PC+HCR), therefore the particular nature of HVO is also not sufficient to explain this behaviour. As such we are forced to conclude that precision is not necessarily a non-decreasing, monotonous function of the observer density and predicting what exactly can affect the performance of the source location estimator is a very complex and, most surely, understudied as of today.

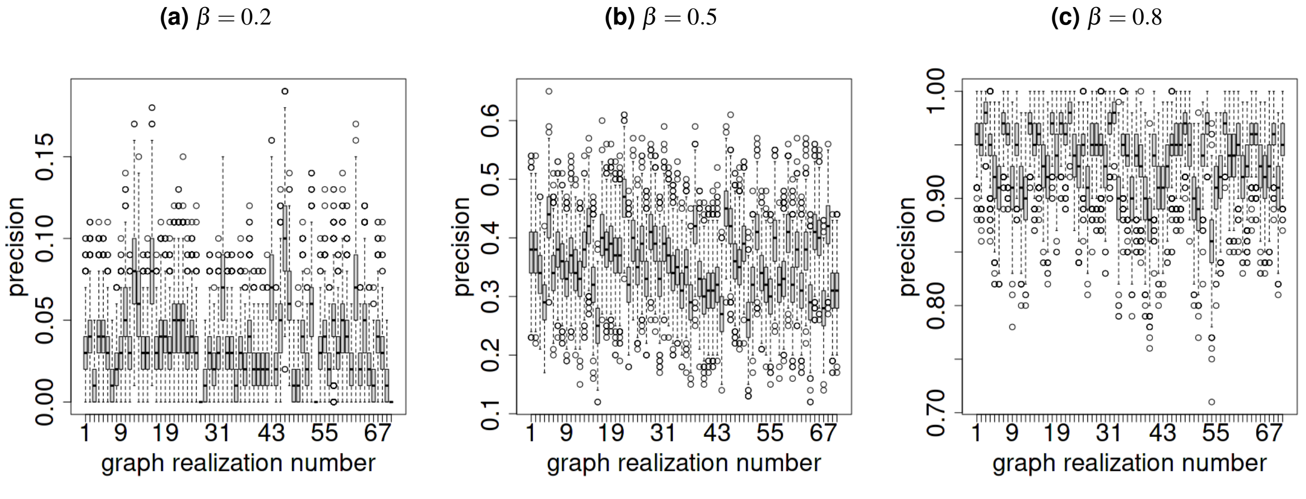

Figure S13. Precision box plots of EPL with HVO (see Methods in main text) for different graph variants. Observer density $d = 0.15$, $\beta \in \{0.2, 0.5, 0.8\}$ (left to right, respectively). Simulations conducted on a Barabási-Albert graph $N = 1000$, $m = 4$. We created 70 different variants of such a graph and performed 100 SI cascades on each, and every box on the plot corresponds to one such variant.

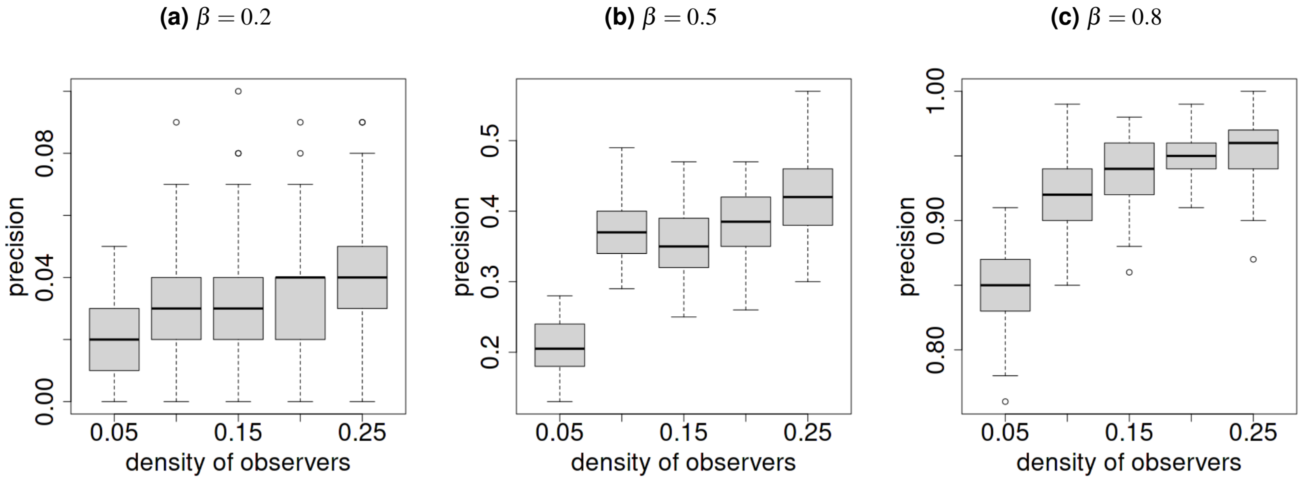

Figure S14. Precision box plots of EPL with HVO (see Methods in main text) for different densities. Each box represents precision from the 70 different realisations (see Fig. S13) of a BA graph ($N = 1000$, $m = 4$). $\beta \in \{0.2, 0.5, 0.8\}$ left to right, respectively.

Results of simulations for ER and BA networks with various average degrees

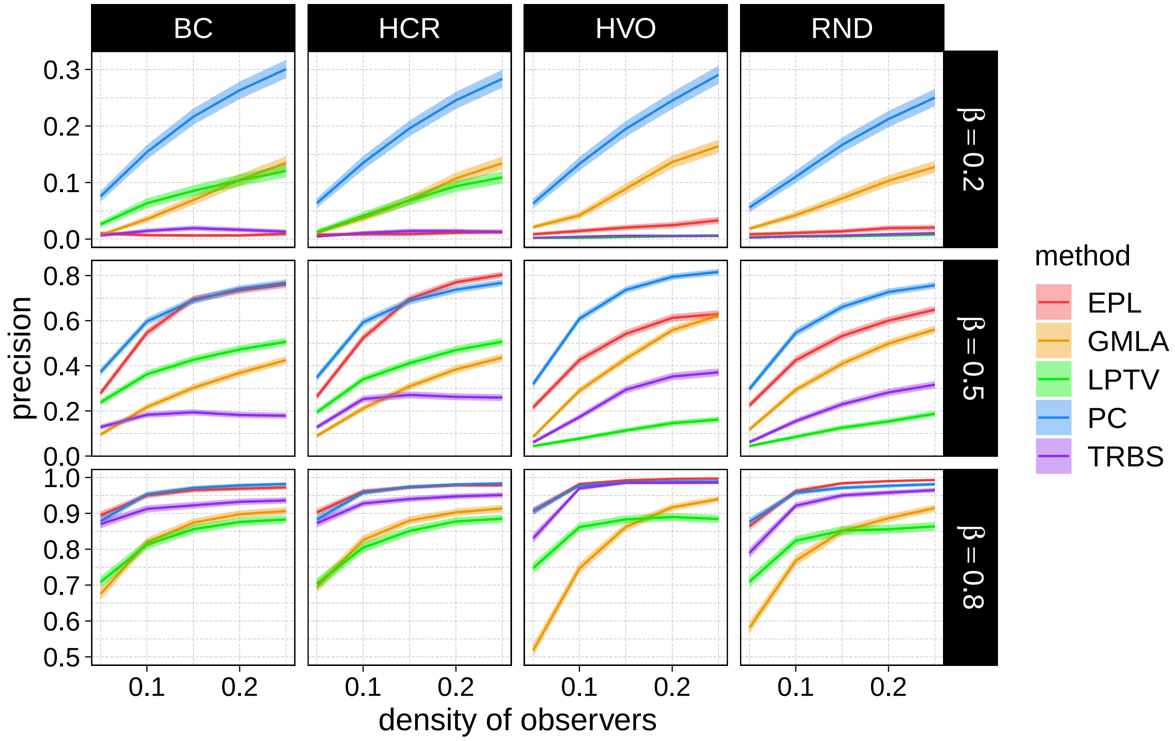

Figure S15. Precision of source localisation on a Barabási-Albert graph ($N = 1000$, $\langle k \rangle = 16$) as a function of observer density, with various observer placements and three different values of infection rate $\beta \in \{0.2, 0.5, 0.8\}$. Each data point is an average of $2 \cdot 10^4$ realisations with error bands representing 5 standard deviations.

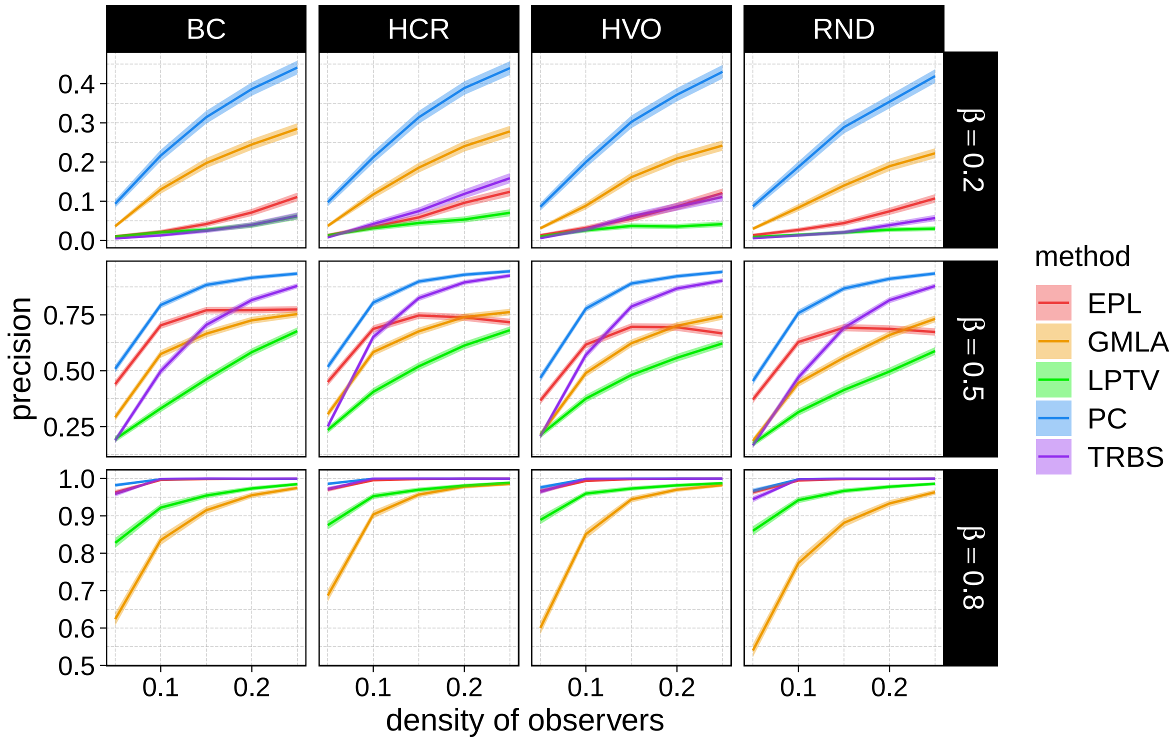

Figure S16. Precision of source localisation on an Erdős-Rényi graph ($N = 1000$, $\langle k \rangle = 16$) as a function of observer density, with various observer placements and three different values of infection rate $\beta \in \{0.2, 0.5, 0.8\}$. Each data point is an average of $2 \cdot 10^4$ realisations with error bands representing 5 standard deviations.

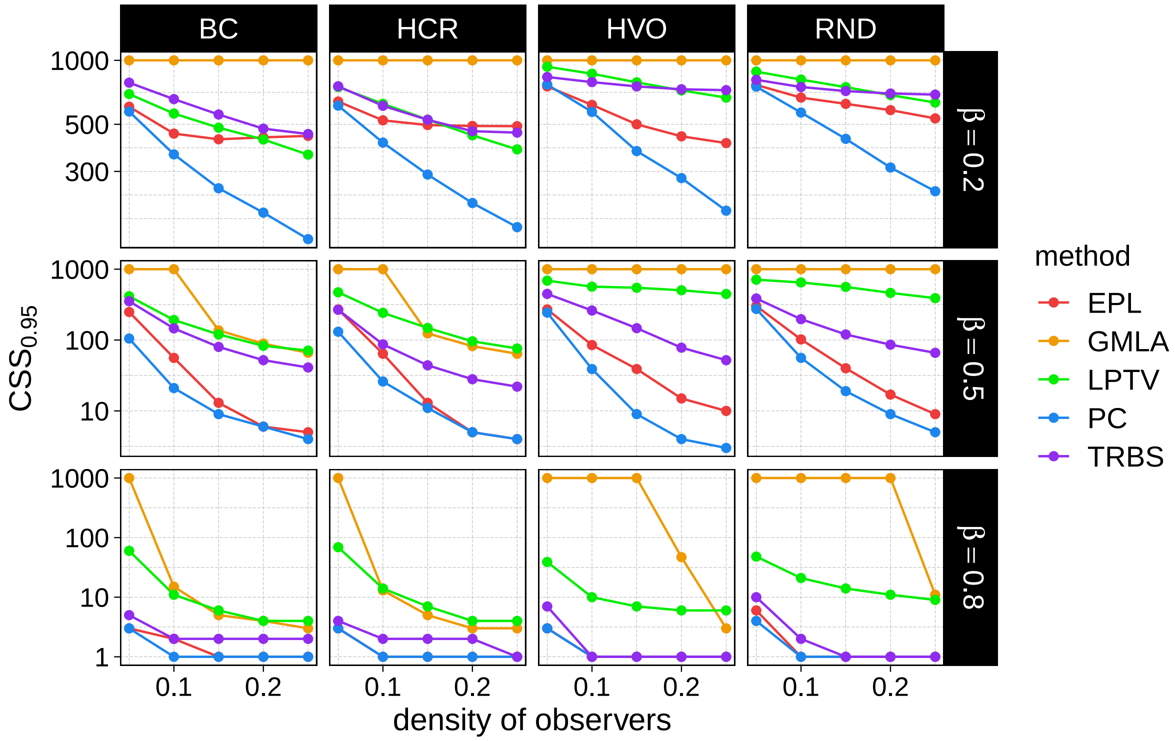

Figure S17. 0.95-CSS of source localisation on a Barabási-Albert graph ($N = 1000$, $\langle k \rangle = 16$) as a function of observer density, with various observer placements and three different values of infection rate $\beta \in \{0.2, 0.5, 0.8\}$, after $2 \cdot 10^4$ realisations.

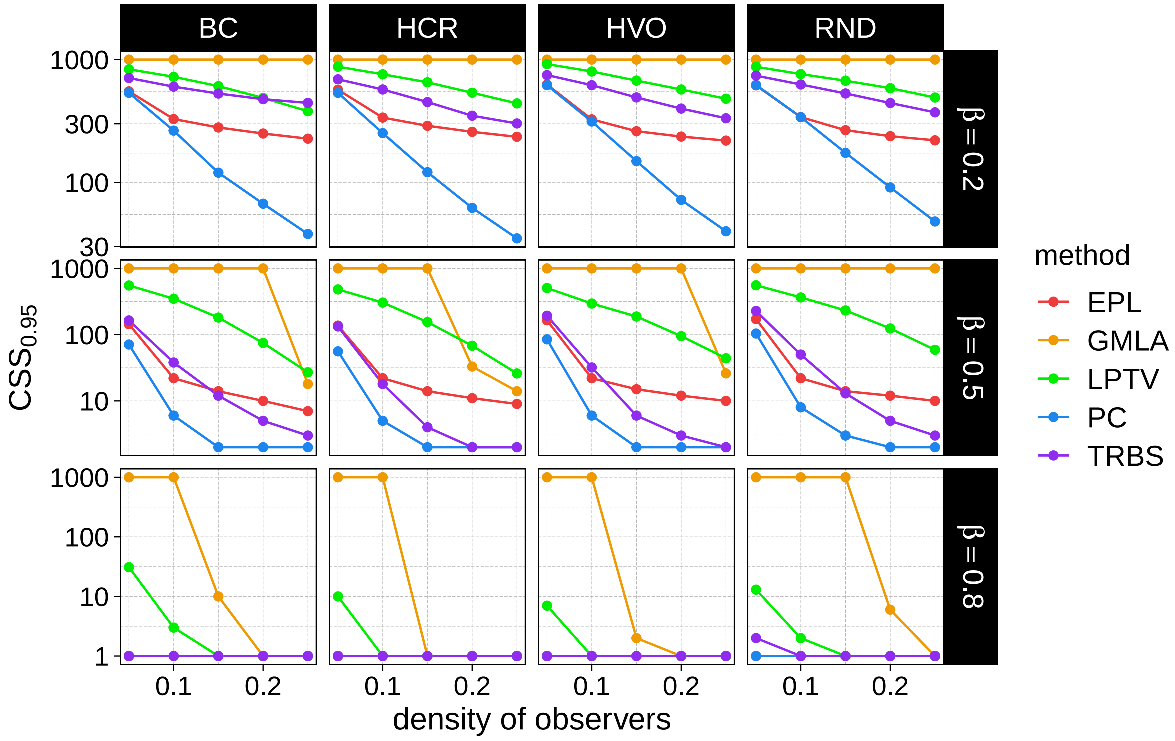

Figure S18. 0.95-CSS of source localisation on an Erdős-Rényi graph ($N = 1000$, $\langle k \rangle = 16$) as a function of observer density, with various observer placements and three different values of infection rate $\beta \in \{0.2, 0.5, 0.8\}$, after $2 \cdot 10^4$ realisations.

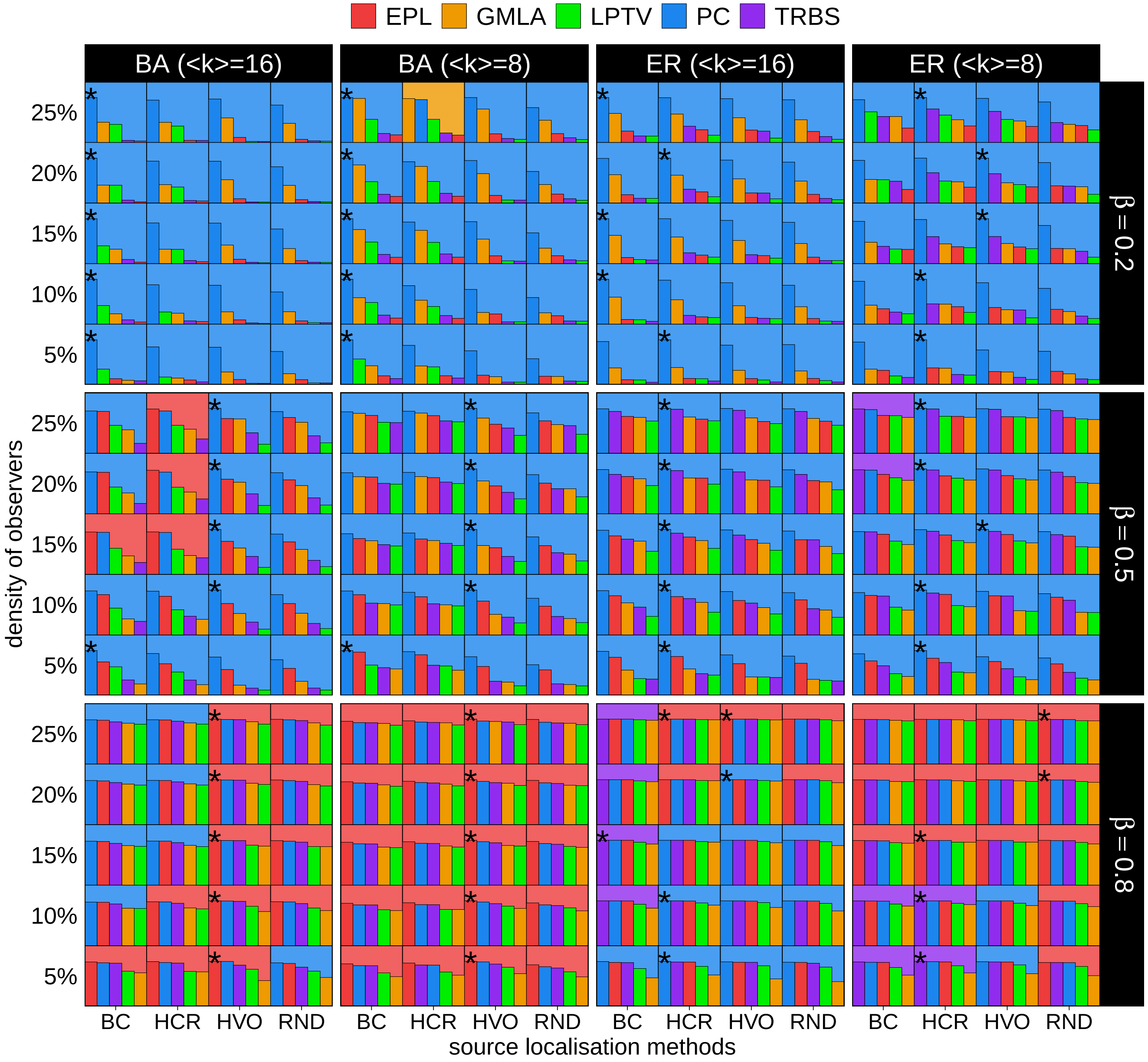

Figure S19. Comparison of precision summary diagrams for network models (Barabási-Albert and Erdős-Rényi) with average degrees 8 and 16. The colours indicate the localisation methods, whereas observer placement strategies are marked per (minor) column in each block (labels are placed at the very bottom of the plot), while minor rows represent observer densities d. Bars within minor blocks show all methods, ordered from the best to the worst (a high precision indicates a high performance), with the background colour of the minor block indicating the best localisation method. The asterisk indicates the best localisation and placement strategy combination per row within a major block, i.e., for a given density, topology and infection rate. Bars are normalised to the highest score per graph, infection rate, and density.

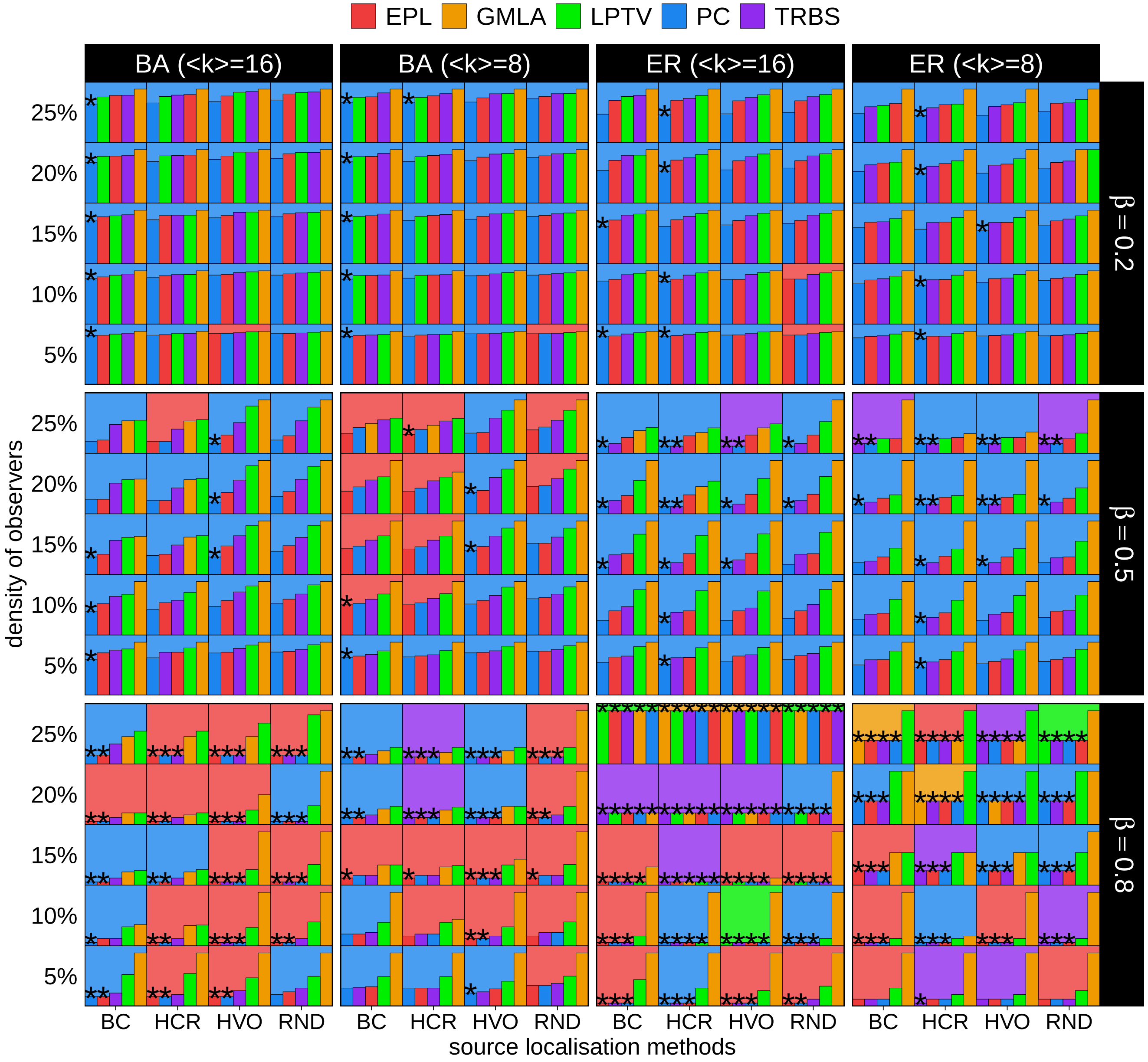

Figure S20. Comparison of 0.95-CSS summary diagrams for network models (Barabási-Albert and Erdős-Rényi) with average degrees 8 and 16. The colours indicate the localisation methods, whereas observer placement strategies are marked per (minor) column in each block (labels are placed at the very bottom of the plot), while minor rows represent observer densities d. Bars within minor blocks show all methods, ordered from the best to the worst (a high precision indicates a high performance), with the background colour of the minor block indicating the best localisation method. The asterisk indicates the best localisation and placement strategy combination per row within a major block, i.e., for a given density, topology and infection rate. Bars are normalised to the highest score per graph, infection rate, and density.

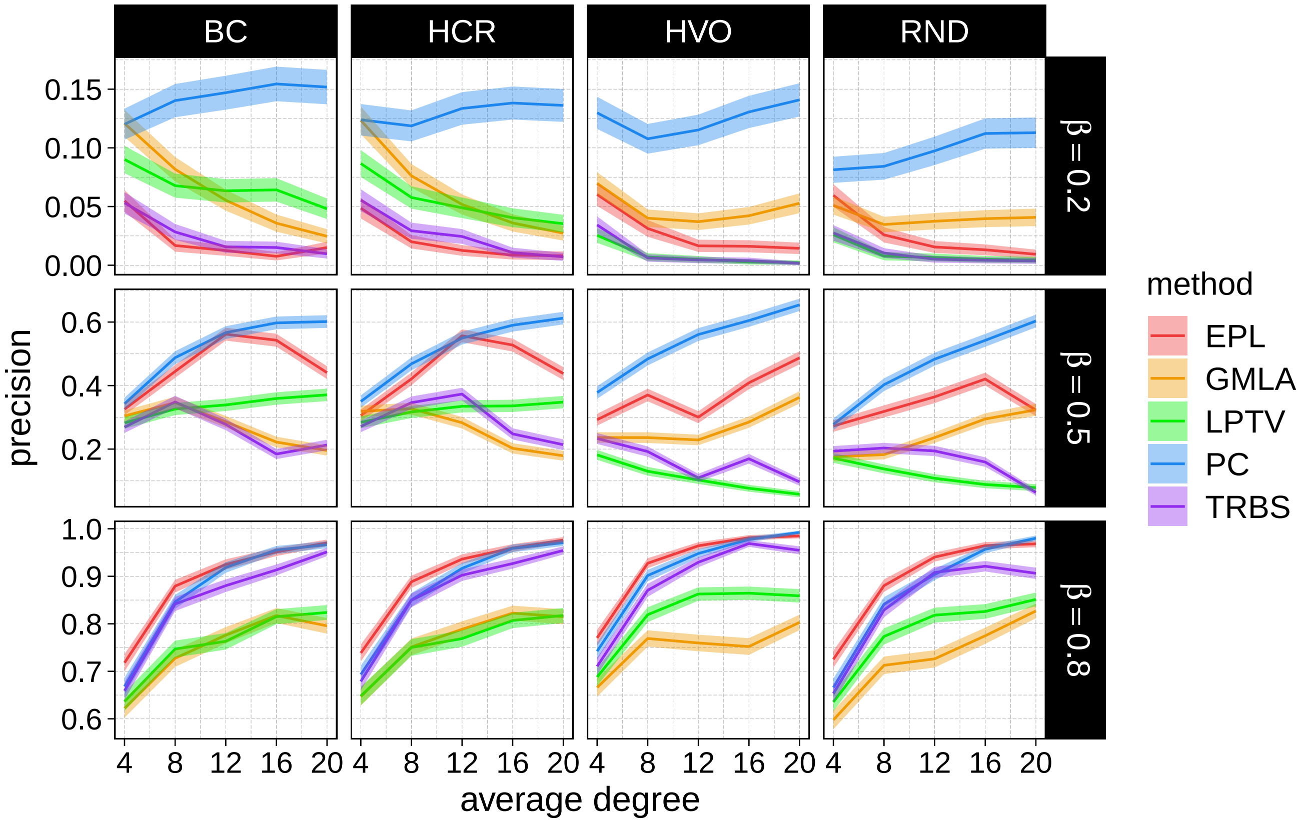

Figure S21. Precision of source localisation on a Barabási-Albert graph ($N = 1000$) as a function of the average degree, with various observer placements (observer density 10%) and three different values of infection rate $\beta \in \{0.2, 0.5, 0.8\}$. Each data point is an average of $1.5 \cdot 10^4$ realisations with error bands representing 5 standard deviations.

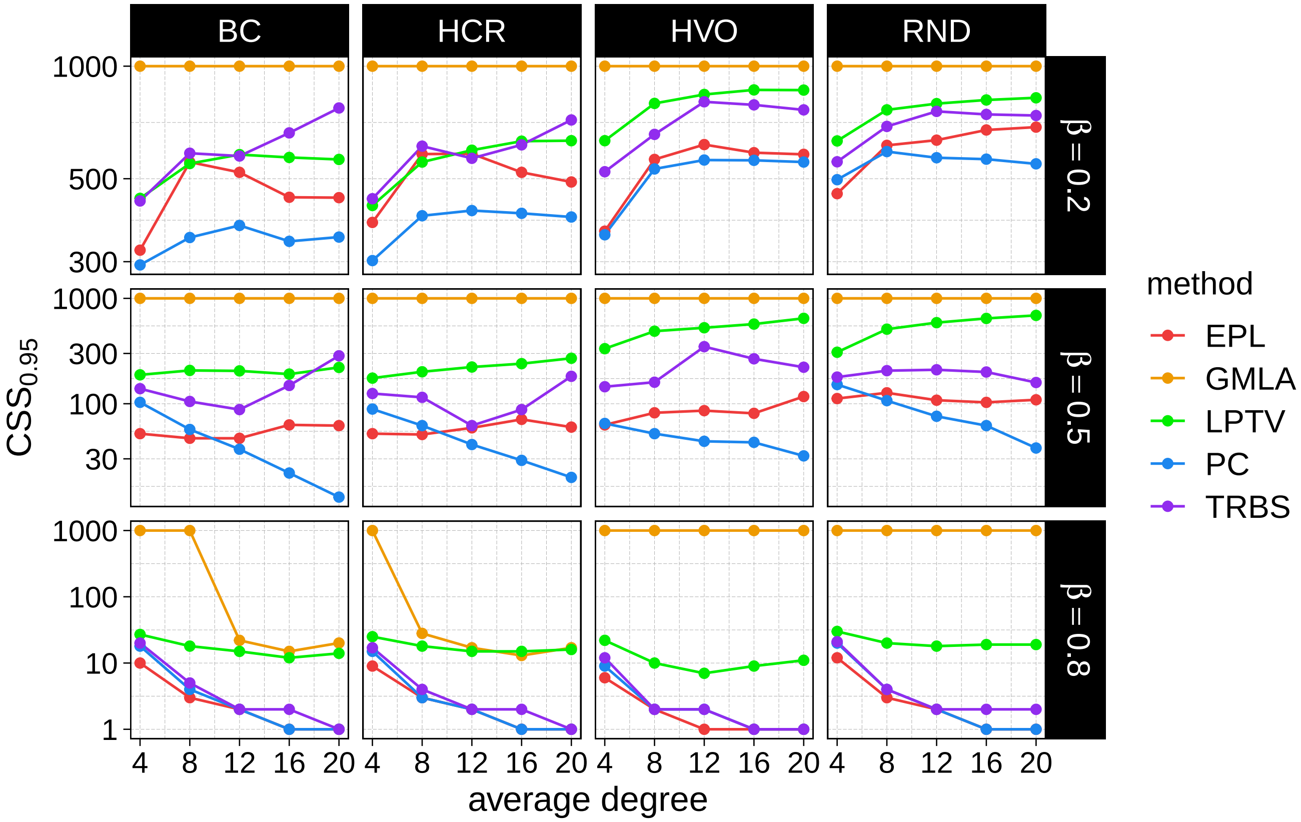

Figure S22. 0.95-CSS of source localisation on a Barabási-Albert graph ($N = 1000$) as a function of the average degree, with various observer placements (observer density 10%) and three different values of infection rate $\beta \in \{0.2, 0.5, 0.8\}$. Each data point is an average of $1.5 \cdot 10^4$ realisations with error bands representing 5 standard deviations.

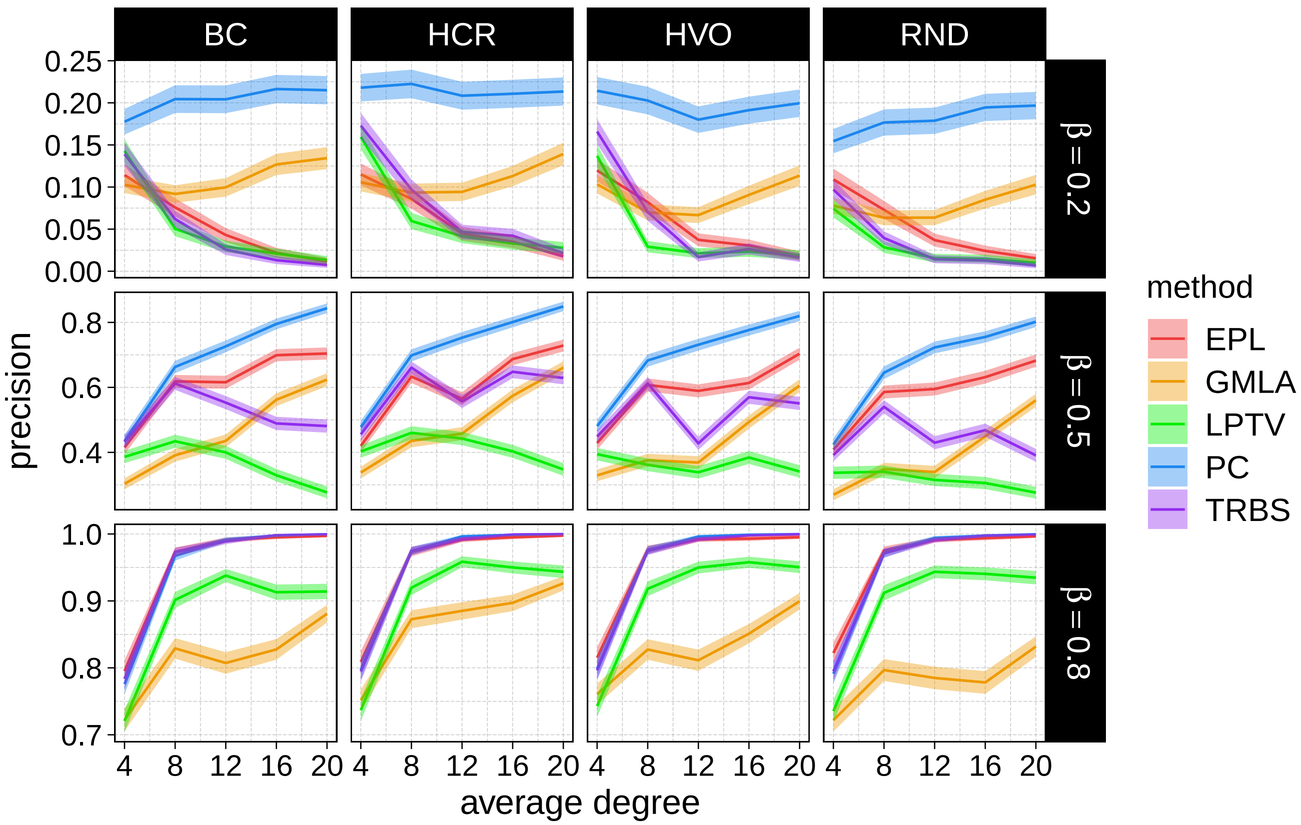

Figure S23. Precision of source localisation on a Erdős-Rényi graph ($N = 1000$) as a function of the average degree, with various observer placements (observer density 10%) and three different values of infection rate $\beta \in \{0.2, 0.5, 0.8\}$. Each data point is an average of $1.5 \cdot 10^4$ realisations with error bands representing 5 standard deviations.

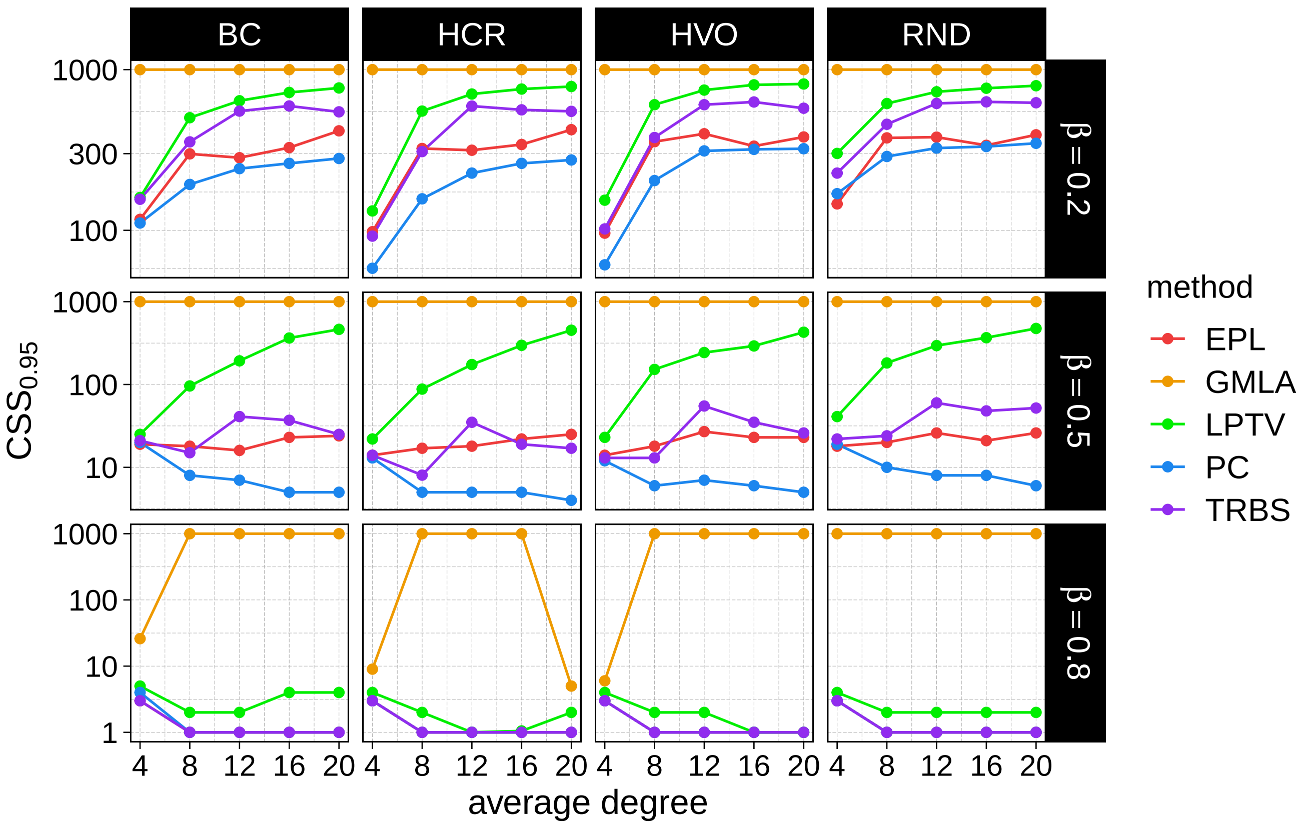

Figure S24. 0.95-CSS of source localisation on a Erdős-Rényi graph ($N = 1000$) as a function of the average degree, with various observer placements (observer density 10%) and three different values of infection rate $\beta \in \{0.2, 0.5, 0.8\}$. Each data point is an average of $1.5 \cdot 10^4$ realisations with error bands representing 5 standard deviations.

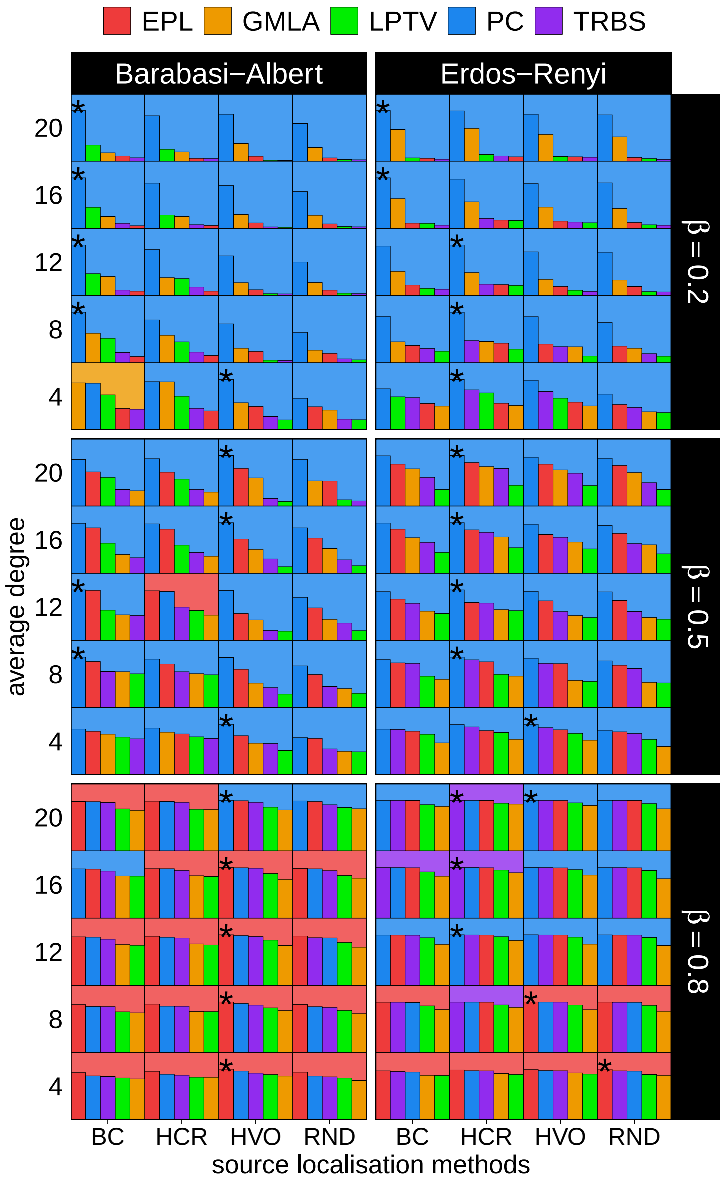

Figure S25. Summary diagrams of precision metric results for synthetic networks (major columns) and infection rates β (major rows). The colours indicate the localisation methods, whereas observer placement strategies are marked per (minor) column in each block (labels are placed at the very bottom of the plot), while minor rows represent the average degree in a network. Bars within minor blocks show all methods, ordered from the best to the worst (a high precision indicates a high performance), with the background colour of the minor block indicating the best localisation method. The asterisk indicates the best localisation and placement strategy combination per row within a major block, i.e., for a given density, topology and infection rate. Bars are normalised to the highest score per graph, infection rate, and density.

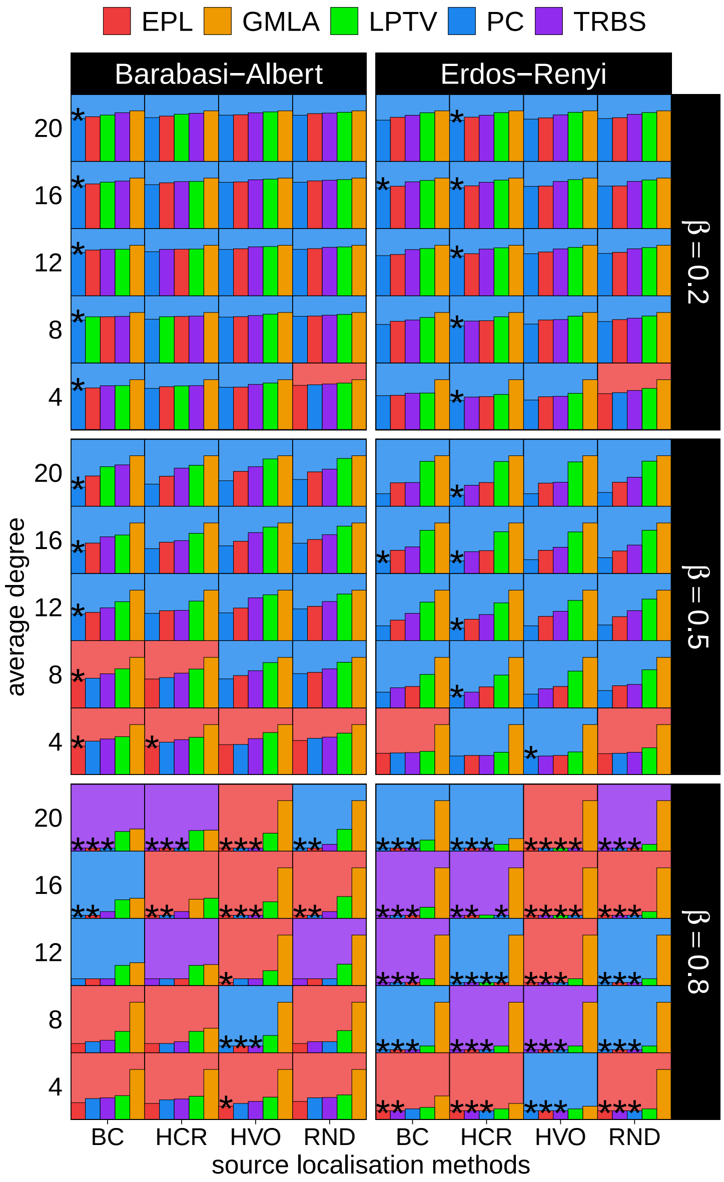

Figure S26. Summary diagrams of 0.95-CSS metric results for synthetic networks (major columns) and infection rates β (major rows). The colours indicate the localisation methods, whereas observer placement strategies are marked per (minor) column in each block (labels are placed at the very bottom of the plot), while minor rows represent the average degree in a network. Bars within minor blocks show all methods, ordered from the best to the worst (a high precision indicates a high performance), with the background colour of the minor block indicating the best localisation method. The asterisk indicates the best localisation and placement strategy combination per row within a major block, i.e., for a given density, topology and infection rate. Bars are normalised to the highest score per graph, infection rate, and density.

Results for a denser grid of infection rate and density of observers 10%

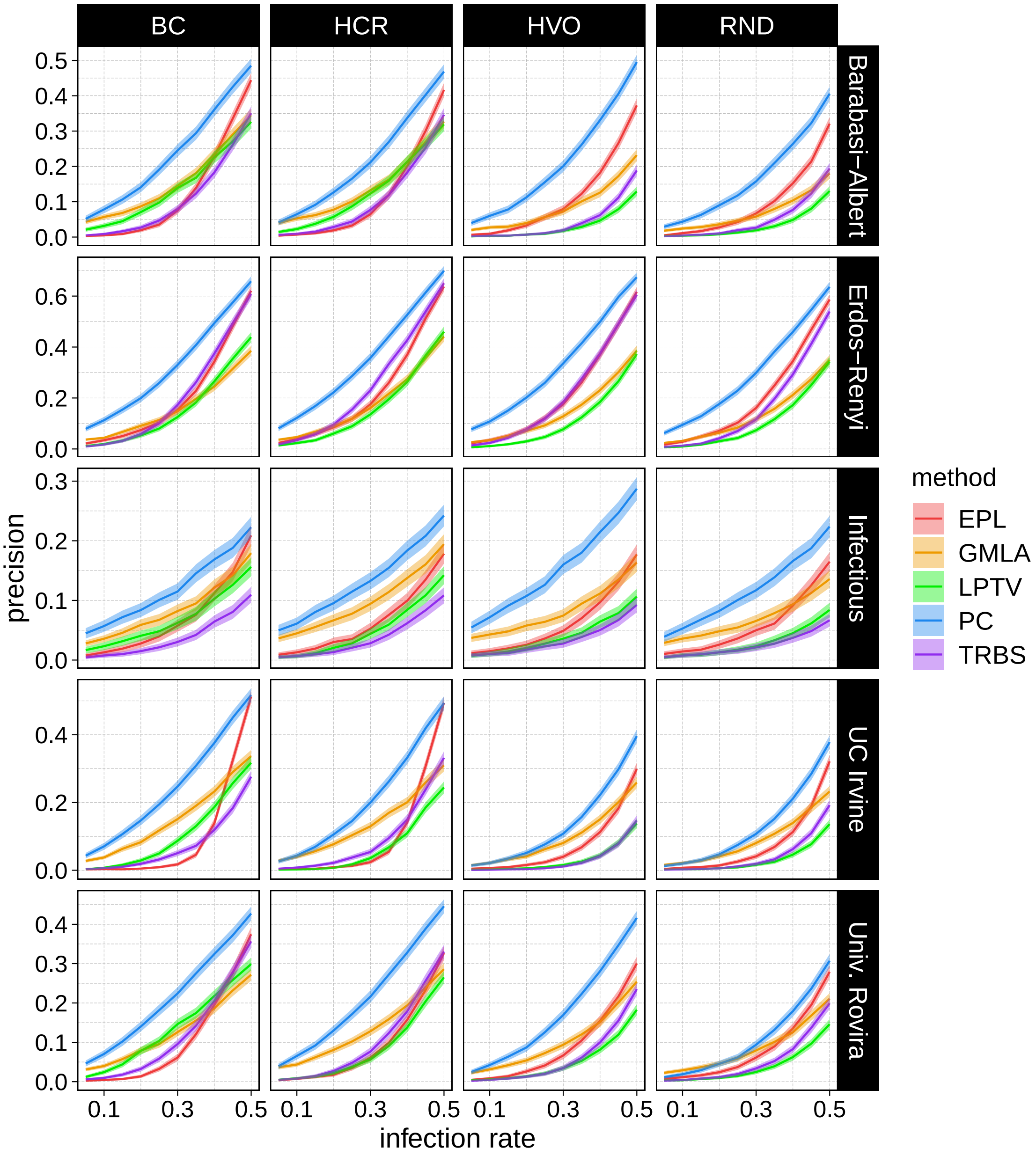

Figure S27. Precision of source localisation for all tested networks (rows) and various observer placements (columns) as a function of the infection rate β. Density of observers is 10% for all cases. Each data point is an average of $1.3 - 1.7 \cdot 10^4$ realisations with error bands representing 5 standard deviations.

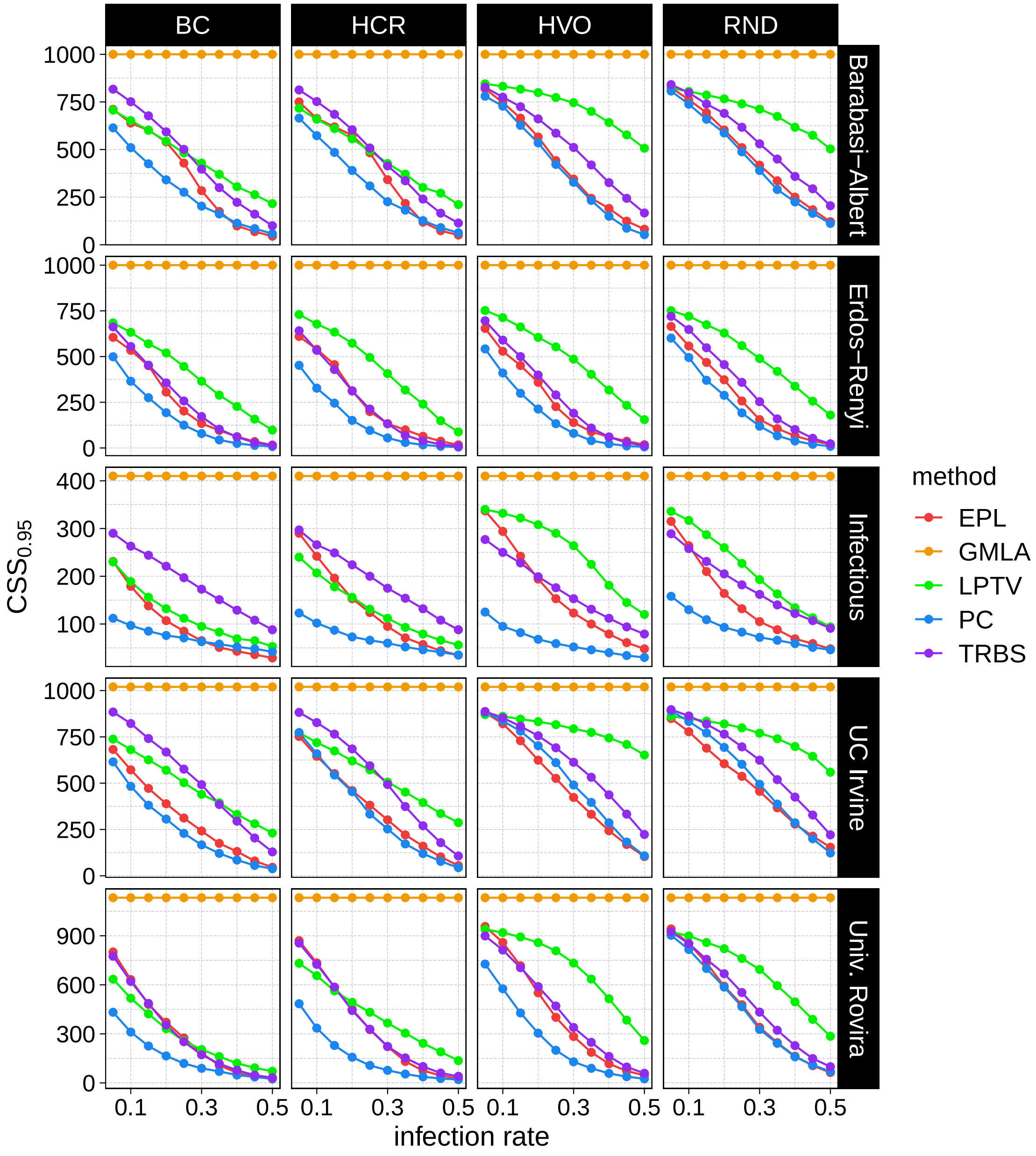

Figure S28. 0.95-CSS of source localisation for all tested networks (rows) and various observer placements (columns) as a function of the infection rate β. Density of observers is 10% for all cases. Each data point is an average of $1.3 - 1.7 \cdot 10^4$ realisations with error bands representing 5 standard deviations.
